# Elucidating the Opportunities and Challenges for Nanocellulose Spinning

**DOI:** 10.1002/adma.202001238

**Published:** 2020-08-23

**Authors:** Tomas Rosén, Benjamin S. Hsiao, L. Daniel Söderberg

**Affiliations:** ^1^ Wallenberg Wood Science Center KTH Royal Institute of Technology Stockholm S‐100 44 Sweden; ^2^ Chemistry Department Stony Brook University Stony Brook NY 11794‐3400 USA

**Keywords:** fiber spinning, flow, nanocellulose, nanostructures, orientation

## Abstract

Man‐made continuous fibers play an essential role in society today. With the increase in global sustainability challenges, there is a broad spectrum of societal needs where the development of advanced biobased fibers could provide means to address the challenges. Biobased regenerated fibers, produced from dissolved cellulose are widely used today for clothes, upholstery, and linens. With new developments in the area of advanced biobased fibers, it would be possible to compete with high‐performance synthetic fibers such as glass fibers and carbon fibers as well as to provide unique functionalities. One possible development is to fabricate fibers by spinning filaments from nanocellulose, Nature's nanoscale high‐performance building block, which will require detailed insights into nanoscale assembly mechanisms during spinning, as well as knowledge regarding possible functionalization. If successful, this could result in a new class of man‐made biobased fibers. This work aims to identify the progress made in the field of spinning of nanocellulose filaments, as well as outline necessary steps for efficient fabrication of such nanocellulose‐based filaments with controlled and predictable properties.

## Graphical abstract

Nanocellulose presents an opportunity for the development of a new class of man‐made biobased fibers that can replace high‐performance fossil‐based fibers, as well as provide fibers with new advanced functionalities. Promising material concepts are available, but successful scale‐up will need more profound insights into the mechanisms that control nanoscale assembly during processing.

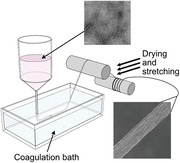

## 1 Introduction

With the global quest for sustainability, there is a strong drive for developing biobased alternatives to today's materials, not only to replace fossil‐based materials but also to provide future materials with new and advanced functionalities. Man‐made fibers are essential in society and are used in various products, directly toward consumers, such as clothes, upholstery, and linens, but also as essential components in aerospace and automotive products as well as in industrial manufacturing processes.

### 1.1 Man‐Made Fibers

In comparison to natural fibers, such as silk‐, cotton‐, or wool‐fibers, man‐made fibers refer to fibers fabricated from either synthetic or natural polymers. Man‐made fibers from synthetic polymers (the largest volume of man‐made fibers) are mainly produced from fossil‐based resources, such as oil or gas. Examples of commonly used polymers are polyamides, polyesters, polyethylene, or polyurethanes. As a result of a century of developments within the area of polymer science and processing, it is currently possible to control the hierarchical structure of synthetic fibers, from the atomistic level up to the fiber level. Thus, one can address the properties and functionalities of the final fibers by developments on all hierarchical levels.

Similar to synthetic fibers, natural fibers are synthesized from the atomic level up to the fiber level. The processes are designed by evolution, generally in the presence of water, to provide sufficient mechanical strength and toughness. Natural fibers are also biodegradable. When fabricating man‐made fibers from natural polymers, the starting point is to use polymers extracted from biological sources, such as cellulose from wood pulp. Hence it is only possible to control the structure can thus only be controlled from the polymer scale and up.

Cellulose represents the starting polymer for the first fabricated man‐made fibers, i.e., Rayon,^[^ ^1^ ^]^ also called regenerated cellulose fibers. In the preparation of regenerated cellulose fibers, highly purified cellulose is subjected to a dissolving process to extract individualized cellulose polymer chains. The biosynthesis in the plant cell wall forms nanofibrils from parallel polymer chains crystallized in a lattice referred to as Cellulose I,^[^ ^2^ ^]^ and the dissolving process breaks the intermolecular bonds to individualize the chains to form a polymer solution. The cellulose polymer solution is spun using a spinneret and precipitated into continuous filaments to form the final regenerated cellulose fiber. The result of the precipitation process and drying is a different crystalline lattice called Cellulose II, which is more thermodynamically stable than Cellulose I. Regarding the mechanical performance as a building block, Cellulose I is significantly stiffer compared to Cellulose II.^[^ ^3^ ^]^ The production of regenerated cellulose fibers depends on biological processes for producing the cellulose polymer since there are no industrial processes capable of producing synthetic cellulose polymer chains.

### 1.2 Spinning Processes

**Figure 1:**
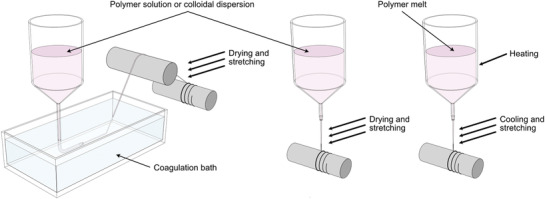
Common means of fiber spinning, wet spinning (left), dry spinning (center), and melt spinning (right).

There are various methods for spinning man‐made fibers based on polymers, either as solutions or melts. **Figure** **1** illustrates three general methods for fiber spinning. The basic principle of the wet spinning process, which also was the first process for spinning man‐made fibers, is to form filaments by extruding a polymer solution through a spinneret (nozzle) into a coagulation or precipitation bath, Figure 1a. After coagulation or precipitation, the extruded filament can be stretched and subjected to various treatments, and after drying, the filament is wound on to a bobbin (cylinder or spindle). In dry spinning (Figure 1b), all the solvent is directly evaporated after extrusion from the spinneret using hot air. During the evaporation process step, it is possible to stretch the extruded filament, which subsequently is wound on to the bobbin. For cases when it is possible to obtain a polymer melt, filaments can be spun by extrusion of the polymer melt that will solidify due to cooling (Figure 1c). As for the other spinning techniques, the filament can be stretched during processing and is wound on to the bobbin. These processes are mainly performed by spinning multiple filaments at the same time by having spinnerets that extrude the solution or melt through multiple holes. Also, even if continuous multifilament bundles are used as is, they are most often further processed into staple fibers, i.e., cut into fibers of discrete length.

### 1.3 Cellulose Filaments from Nanocellulose

Extraction of the nanocellulose has been possible for several decades, and it is today possible to obtain larger quantities produced in industrial scale, both as cellulose nanocrystals (CNC) and as cellulose nanofibers (CNF) of different types. These are generally produced from wood‐pulp but can be produced from underutilized nonwood plants such as agriculture residues and invasive species. Also, there is bacterial nanocellulose, which shares many properties with the plant‐based nanocelluloses. However, the morphology and behavior of extracted nanocelluloses vary with origin and processing protocols, and it is challenging to produce nanocelluloses with predetermined and constant properties.

By using nanocellulose, it is possible to fabricate materials that use Cellulose I as a building block, which is not possible with regenerated cellulosic materials.^[^ ^4^ ^]^ Thus, there seems to be a potential for spinning filaments from nanocellulose that would have better mechanical performance compared to regenerated cellulose filaments. Furthermore, nanocellulose spinning is mainly water‐based, which means that it is possible to add components that would be affected by the solvents used for the spinning of regenerated fibers.

Here, we present a review of the present state‐of‐the art related to spinning of nanocellulose filaments. Our focus is CNF‐based filaments, but some relevant results obtained with cellulose nanocrystals (CNC) and bacterial cellulose have been included. Knowing that CNF today is used as an acronym for cellulose nanofibers, we have chosen to use the term nanofibril throughout to indicate that we discuss nanoscale objects such as CNF and CNC that are subjected to the same mechanisms as other nanofibrillar materials.^[^ ^5^ ^]^ We have also tried to identify what controls nanoscale interactions and self‐assembly, as well as how this affects the hierarchical structure of nanocellulose filaments. Finally, we have identified what we believe is the logical path forward, including the need for more systematic characterization of nanocelluloses and process conditions in order to fabricate a new generation of advanced nanocellulose‐based materials with predictable and repeatable properties.

## 2 Spinning of Nanocellulose Filaments

### 2.1 Wet Spinning by Syringe Extrusion

**Figure 2:**
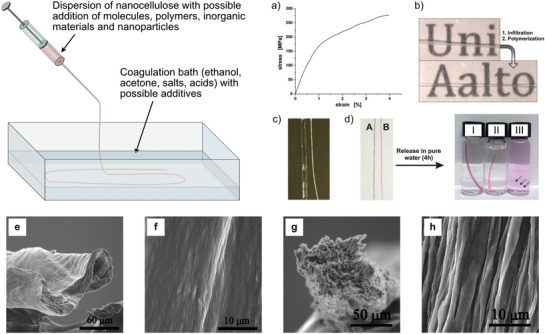
The principle of syringe‐based wet‐spinning of a CNF‐dispersion (top left). a–d) Mechanical properties, optical transparency, wettability control, and encapsulation/release of model compounds for wet‐extruded CNF filaments.^[^ ^7^ ^]^ a) Representative stress–strain curve of a filament. b) Opaque as‐prepared filaments can be rendered transparent by impregnation with a resin matching the refractive index of constituent CNF. c) Filaments after swelling in water for 4 h (no visual change after 3 weeks), before (left) and after (right) surface modification with a fluorinated silane. d) Filaments loaded with: A) hydrophobic nile red and B) hydrophilic rhodamine 6G. Different release characteristics for: I) hydrophobic nile red, II) cationic rhodamine 6G, and III) anionic sulforhodamine. Anionic sulforhodamine is released from the anionic CNF. The arrows indicate the swollen CNF filament in (V). e–h) Representative SEM images of wet‐spun filament cross‐sections and filament surfaces.^[^ ^6^ ^]^ e,f) Filament cross‐section (e) and surface (f) spun from wood‐pulp CNF. Filament cross‐section g) and surface h) spun from tunicate CNF. a–d) Adapted with permission.^[^ ^7^ ^]^ Copyright 2011, American Chemical Society. e–h) Adapted with permission.^[^ ^6^ ^]^ Copyright 2011, American Chemical Society.

The first published paper describing the wet spinning of CNF obtained by 2,2,6,6‐tetra‐methylpiperidinyl‐1‐oxyl (TEMPO)‐mediated oxidation (TCNF) into filaments extracted from wood‐pulp and tunicate^[^ ^6^ ^]^ used a straightforward wet spinning approach, where the spinning dope, i.e., a TCNF dispersion at 1 wt% concentration was extruded through a syringe needle with an inner diameter of 0.95 mm into an acetone coagulation bath (**Figure** **2**). The experiments were performed at different extrusion rates. The results showed that with respect to microscale and mesoscale structures, filaments spun from wood‐pulp TCNF exhibited irregular cross‐sections with relatively smooth surfaces (Figure 2e,f), which became hollow and collapsed at high spinning rates. In comparison, tunicate filaments had more symmetric cross‐sections and a distinct surface structure with ridges aligned in the spinning direction (Figure 2g,h).

As a means of characterizing the internal nanostructure of the filament, the orientation index was extracted from wide‐angle X‐ray scattering (WAXS) data, where the coupling between mechanical performance and nanostructure alignment of wood‐pulp TCNF could be found. Typically, the filaments showed an increased alignment with increasing spinning rate, resulting in improved mechanical tensile performance, i.e., Youngs’ modulus and tensile strength, and reduced strain‐at‐break ratio. For wood pulp filaments, the maximum strength was in the range of 320–330 MPa, stiffness in the range of 19–24 GPa and strain‐at‐break ratio around 3%. A similar but less obvious correlation was found for tunicate filaments, where the strongest and stiffest filament exhibited a lower orientation index with a similar strain‐at‐break ratio. However, compared to wood pulp TCNF, the filament properties obtained with tunicate TCNF were significantly better, giving a strain‐at‐break ratio of ≈6% and strength of 406 MPa, but with similar stiffness of 19 GPa. It was noted that the carboxylate content of the two nanocelluloses were quite different, where the wood‐pulp TCNF had a higher surface charge. These results clearly showed that the spinning of nanocellulose filaments, using a simple and nonoptimized set‐up, could produce filaments that have competitive mechanical properties in comparison to cotton and lyocell fibers, specifically regarding the Young's modulus.

Based on the syringe‐based spinning process for fabricating continuous filaments from wood‐pulp TCNF, the possibility of introducing additional functionality was studied using, e.g., ethanol, dioxane, or isopropyl alcohol as the coagulation bath.^[^ ^7^ ^]^ It was shown that filaments can be fabricated with more cylindrical cross‐sections, and that they could be nanoporous. This was presented as a platform for functionalization through infusing the filaments with functional components/additives. As a demonstration, it was shown that the infusion of a suitable index‐of‐refraction matched matrix, the filaments became transparent (Figure 2a–d). Furthermore, it was shown that filaments could be made electrically conducting as well as magnetic. The mechanical performance was similar to what had been obtained earlier,^[^ ^6^ ^]^ with a Young's modulus of 22.5 GPa, tensile strength of 275 MPa and strain‐at‐break ratio of 4%. It was also pointed out that the postdrawing/stretching process could be a viable route to increase the TCNF alignment leading to improved mechanical properties.

**Figure 3:**
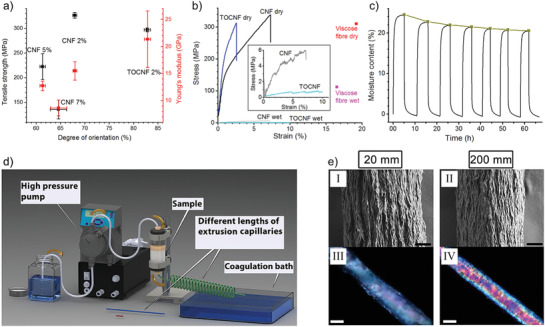
a–c) Effect of spinning concentration and humidity cycling on wet‐spun filaments from CNF and TCNF (=TOCNF).^[^ ^8^ ^]^ a) Filament tensile strength and Young's modulus as a function of the degree of orientation. Error bars correspond to the standard deviation among the tested specimens. b) Representative stress–strain curves for CNF 2% and TCNF 2% filaments in dry condition and after soaking in water for two hours. The drastic reduction in strength illustrates the deleterious effect of water. As a reference, tensile strength and elongation for viscose fiber (coarseness 0.17 g km^−1^, diameter ≈6 μm) are included. The inset shows the profiles in wet state using reduced plot scales. c) Water sorption analysis for CNF 2% filament. The curve passing through the square symbols, which represents the development of the equilibrium moisture content (EMC) at RH 95%, is added to guide the eye. d,e) Effect of spinning using different capillaries (length and diameter) and flow‐rates.^[^ ^9^ ^]^ d) The scheme of the spinning device used to investigate the effect of capillary length, diameter, and flow‐rate. e) Images of the surface topology of filaments showing an increasing smoothness/orientation of surface structures with capillary length (I,II), and optical birefringence of same fibers illustrating pronounced changes in the birefringence (III,IV). a–c) Adapted under the terms of the CC‐BY Creative Commons Attribution 4.0 International license (https://creativecommons.org/licenses/by/4.0).^[^ ^8^ ^]^ Copyright 2016, The Authors, published by Springer Nature. d,e) Adapted under the terms of the CC‐BY Creative Commons Attribution 4.0 International license (https://creativecommons.org/licenses/by/4.0).^[^ ^9^ ^]^ Copyright 2017, The Authors, published by Springer Nature.

By using nonmodified wood‐pulp CNF and a syringe‐based spinning set‐up, the effect of concentration on the filament properties was further elucidated.^[^ ^8^ ^]^ The results were also compared to the spinning of a wood‐pulp TCNF as a reference. The maximum strength of 326 MPa and a Young's modulus of 15.5 GPa was obtained for the filament spun from a CNF dispersion with 2% concentration, where the filament spun from the TCNF dispersion (same concentration) exhibited lower strength but higher stiffness, i.e., 297 MPa and 21.3 GPa, respectively (**Figure** **3**a). It was hypothesized that interfibrillar contacts are an important factor with respect to the ability to control the nanofibril alignment during spinning. As a result, the concept of crowding factor was adopted (see further discussion in Section 4.1) to explain the observation. In specific, two mechanisms for nanofibril alignment were discussed: i) flow‐induced alignment caused by hydrodynamic extensional and shear forces, and ii) contact‐induced alignment, induced by the packing of nanofibrils toward an oriented structure upon solvent depletion. Furthermore, they investigated the effect of elevated and cycling of humidity (95%RH) on dry filaments and showed that the mechanical performance of CNF‐filaments deteriorates significantly in comparison to viscose. In addition, the negative effects were found to be more pronounced for TCNF than for nonmodified CNF (Figure 3b). Finally, the “hornification” process was quantified by studying the equilibrium moisture content progression with humidity cycles spotted. The equilibrium moisture content for the 2% CNF was about 20% at 95%RH (Figure 3c).

In another study, it was found that the relation could be broken by spinning a nonmodified CNF at 2% concentration though capillaries with different lengths and flow‐rates, (Figure 3d).^[^ ^9^ ^]^ Longer capillaries and higher flow‐rates resulted in filaments with an increase in strength, and strain‐at‐break ratio, thus an increase in toughness. Two hypothesizes where presented to explain how strength and stiffness could be increased without the reduction in strain‐at‐break. One hypothesis was that shear forces induced alignment in the capillaries before coagulation, in a state where the nanofibrils were free‐flowing, which would counteract postspinning stretching (Figure 3e). This process would enhance defects within the network and cause rupture due to tensile strain. The second hypothesis was that the CNF used in the study had a relatively high hemicellulose content, which could affect the filament properties, similar to the effect of hemicellulose in wood‐based fibers.

The effect of the coagulation stage has been further elucidated by wet spinning of wood‐pulp TCNF using different spinning baths comprising of organic nonsolvents (ethanol or acetone) or electrolyte solutions with NaCl, CaCl_2_, or HCl.^[^ ^10^ ^]^ Enhanced interfibrillar interactions were observed after solvent exchange (ethanol and acetone antisolvents) or via electrostatic screening (via monovalent cations), complexation (aqueous electrolytes) and ionic crosslinking (via multivalent cations). The results showed clear effects between coagulant type and structural and mechanical properties of the spun filaments as well as thermal stability.

It has also been demonstrated that it is possible to fabricate CNF‐filaments as a core–shell material.^[^ ^11^ ^]^ Using the wet spinning process and different coagulation baths it was possible to fabricate core–shell structures using guar gum or cellulose acetate. The structure was obtained by spinning using two syringes connected to a coaxial needle. The filaments with cellulose acetate as the shell showed significantly better water absorption capacity (43 g water g^−1^ dry filament), which was attributed to reduced densification of the CNF core structure, which results in filaments with a high accessible area and more pores.

### 2.2 Wet Spinning by Flow Focusing

**Figure 4:**
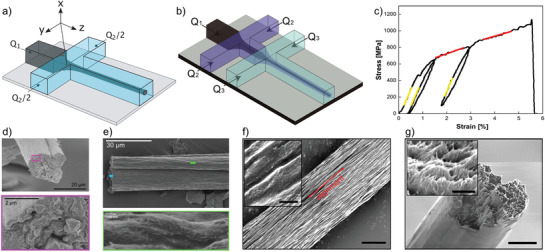
a,d,e) Single flow‐focusing spinning.^[^ ^13^ ^]^ a) Flow‐focusing channel with *Q*_1_ representing the core flow of CNF dispersion and *Q*_2_ representing the sheath‐flows with Na^+^ to induce coagulation. In (d) the cross‐section of a spun filament can be seen, in (e) the surface structure. b,c,f,g) Double flow‐focusing spinning^[^ ^17^ ^]^ where (b) shows the geometry where coagulation is induced by the second step, *Q*_3_, whereas *Q*_2_ is deionized water. c) A stress–strain curve for a filament and all yellow and all red lines have identical slope. f) SEM image of the fiber surface with anisotropic arrangement, scale bar is 3 μm and 400 nm in the inset. g) SEM image of the cross section of the fiber, scale bar is 3 μm. a,d,e) Adapted under the terms of the CC‐BY Creative Commons Attribution 3.0 Unported license (https://creativecommons.org/licenses/by/3.0/).^[^ ^13^ ^]^ Copyright 2014, Springer Nature. c,f,g) Adapted with permission.^[^ ^17^ ^]^ Copyright 2018, American Chemical Society.

As an alternative to the syringe‐based wet spinning process, the concept of flow‐focusing^[^ ^12^ ^]^ was adopted as a means to spin CNF into filaments.^[^ ^13^ ^]^ Instead of spinning a dispersion into a coagulating bath, the core flow of a flow‐focusing device, consisting of a nanocellulose dispersion, is formed into a more or less cylindrical thread by the so‐called sheath flows, which also induce coagulation (**Figure** **4**a). As a starting material, wood‐pulp carboxymethylated CNF was used and coagulation was induced through a surface‐charge‐controlled gel transition^[^ ^14^ ^]^ using Na^+^ ions in the sheath flow. The results showed that the nanofibril alignment could be enhanced by controlling the elongational flow induced in the device, making it clearly different from a shear‐alignment approach. Apart from the flow‐field, the flow‐focusing spinning differs from syringe‐based spinning in that the spinnable CNF concentration can be very low, in the range of 0.2–0.3 wt%. With the low concentration, appropriated dimensions of the channels (1 mm) and suitable volumetric flow‐rates of the core and sheath flows, the resulting filaments exhibited an almost cylindrical cross‐section with diameter of 22–38 μm, having a surface structure with ridges aligned along the filament direction (Figure 4d,e). It was found that the flow‐rate relation could be used to control the CNF alignment without the need to change the channel geometry. Mechanical tensile performance of the filament showed the maximum tensile strength of 490 MPa with a Young's modulus of 17.6 GPa and a strain‐at‐break ratio of 6.4%. From the stress–strain curves, the toughness was estimated to be around 18 MJ m^−3^.

A double flow‐focusing device was introduced to further improve the flow‐focusing process (Figure 4b).^[^ ^15^ ^]^ In this device, the first flow‐focusing flow involves pure water that primarily detaches the CNF dispersion from the solid surfaces of the channel, while the second step creates the elongational flow‐field and introduces the coagulation agent. This set‐up offers the possibility of adding multiple components sequentially to create filaments of different core–shell structures, thus enhances the flexibility of the device to spin varying types of CNF dispersions since the coagulation process would never occur on the solid surface.

The double flow‐focusing set‐up was later employed to further improve the mechanical properties of CNF‐based filaments by tuning and modifying the CNF dispersion. For example, carboxymethylated wood‐pulp CNF was added with 10% recombinant spider silk proteins to produce the spinning suspension, while the sheath flow using a HCl solution was applied as a coagulant instead of a NaCl solution.^[^ ^16^ ^]^ The shift from Na^+^ to H^+^ reduced the time to achieve gelation downstream of the second sheath flow due to the increased diffusion rate. For the pure CNF‐based filaments, a strength of 830 MPa, stiffness of 53 GPa and a strain‐at‐break ratio of 6% was achieved, resulting in a toughness of about 37 MJ m^−3^. When considering the addition of the spider silk proteins, the filament properties (strength, stiffness and strain‐at‐break) increased to 980 MPa, 54 GPa, 10%, and 55 MJ m^−3^, respectively.

In a later study, wood‐pulp TCNF was used to investigate the effects of surface‐charge (also causing different mean nanofibril lengths) and chemical crosslinker on the resulting filament properties.^[^ ^17^ ^]^ A set of four CNF dispersions were evaluated having surface charge in the range of 550−1360 μeq g^−1^. These samples were carefully characterized with respect to fiber length and width distributions. Filaments were spun using the double flow‐focusing set‐up and it was found that the combined effect of surface charge and nanofibril morphology had a distinct effect on the mechanical properties. Furthermore, it was shown that the strength and stiffness improved at the expense of reduced strain‐at‐break ratio, when applying 1,2,3,4‐butane tetracarboxylic acid (BTCA) as a crosslinker or when tests were performed at lower relative humidity. Pure CNF‐filaments showed a maximum Young's modulus of 70 GPa and strength of 1200 MPa. By replacing HCl in the second sheath flow with BTCA, the strengths increased to 1430 MPa after heat‐treating the spun filaments at 105 °C for 1 h. During the cyclic loading of filaments, the stress–strain results showed that the elastic modulus did not change even when the filament was subjected to the plastic deformation (Figure 4c, where the slopes of the yellow and red lines remained the same after cycling of the load). It is interesting to note that the resulting filaments have an almost perfectly circular cross‐section but the surface was covered in ridges aligned along the filament direction (Figure 4f).

It has recently also been shown that flow‐focusing can be used to fabricate filaments from cellulose nanocrystals (CNC),^[^ ^18^ ^]^ where he double flow‐focusing set‐up was operated with HCl as the second sheath flow. Dispersions of carboxymethylated wood‐pulp CNF and wood‐pulp CNC prepared by sulfuric acid hydrolysis were used as the core flow, as well as a CNC/CNF blend. In order to successfully spin the CNC dispersion, the concentration had to be 1.3 wt% compared to 0.3% for the CNF. Produced CNC filaments had inferior mechanical properties compared to the CNF filaments, but it was suggested that by being able perform microfluidic spinning with CNC, which has a different morphology compared to CNF, it should be possible to adapt the processes for many other high‐aspect‐ratio nanoparticles.

### 2.3 Dry Spinning

As an alternative to spin nanoscale CNF into microscale filaments using coagulation, it is possible to spin it without the coagulation step, i.e., dry spinning.^[^ ^19^ ^]^ In a demonstration process, a high‐concentration unmodified CNF from banana rachis, an agriculture residue, was spun directly without the coagulation step. It was found that dry spinning could only be achieved when the CNF concentration exceeded 6.5 wt%. This is a much simpler spinning process compared to the processes based on sol–gel transition. However, the process does not provide high individual mobility for nanocellulose fibrils to reorganize themselves to maximize the interfibrillar interactions (i.e., van de Waals and hydrogen bonding forces). As a result, the spun filaments exhibited reasonably cylindrical cross‐section but the mechanical properties were only half when compared to those from filaments produced by wet spinning.^[^ ^20^ ^]^

### 2.4 Interfacial Complexation Spinning

A totally different process for filament spinning and functionalization is interfacial polyelectrolyte complexation.^[^ ^21^ ^]^ This fabrication route generates filaments by self‐assembly induced at the interface of two overlapping drops of different polyelectrolyte solution, one being a polycation solution and the other a polyanion solution. The self‐assembled film forming at the interface can be picked up by a pair of tweezers, whereby a continuous filament is formed. This spinning technique has been used to fabricate filaments from various biological sources.^[^ ^22^ ^]^ Apart from being a novel fabrication process, it is argued that it gives rise to a characteristic nervation/veining pattern on the surface and oriented along the length of filaments, corresponding to sub‐filaments throughout the drawn filament that has a sub‐micrometerdiameter. This substructure of the filaments can be readily proven by fanning‐out the filament before drying. It should be noted that these structures are very similar to what is obtained using other spinning techniques (Figures 2h,3e, and 4f).

**Figure 5:**
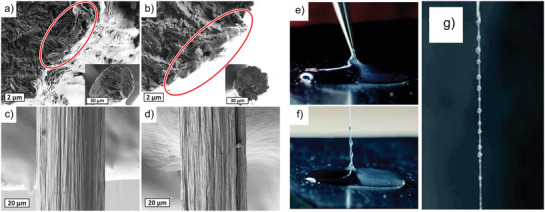
a–d) SEM images of filaments formed through interfacial complexation.^[^ ^23^ ^]^ a,b) Close‐ups of the cross‐sectional fracture surface of individual a) TCNF/Chitosan and b) TCNF/PDADMAC filaments. Insets in both images show the nearly circular shape of the cross‐section. Notice the encircled thin skin formed by the cationic polymers, now folding on the fracture surfaces. c,d) Side‐views of TCNF/chitosan and TCNF/PDADMAC filaments respectively (stretched 20% prior to drying), which show the veining/nervation pattern of the surface. e–g) Drawing of filaments through interfacial complexation.^[^ ^24^ ^]^ e) Formation of the interface between the TCNF suspension (clear liquid) and chitosan solution (cloudy liquid), f) gently pulling the viscous thread from the interface, and g) image of the filament taken immediately after wet‐drawing. a–d) Adapted with permission.^[^ ^23^ ^]^ Copyright 2017, American Chemical Society. e–g) Adapted with permission.^[^ ^24^ ^]^ Copyright 2017, The Royal Society of Chemistry.

By replacing the polyanion with negatively charged wood‐pulp TCNF, and using polydiallyldimethylammonium chloride (PDADMAC) or cationic chitosan as the polycation, the interfacial complexation strategy was shown to be applicable also for fabricating nanocellulose filaments.^[^ ^23^ ^]^ It was demonstrated that filaments could be fabricated for a wide range of concentrations, and it was suggested the interface that is formed between the two components provides a strong load‐bearing skin, which also blocks diffusion of the polycation into the filament, resulting in a core–shell structure. The typical nervation/veining pattern could be seen on the surface of the filaments (**Figure** **5**a–d), and the mechanical properties were shown to be in the same range as for wet‐spun filaments CNF filaments with a tensile strength of almost 250 MPa and a Young's modulus of ≈20 GPa. By drawing another filament in parallel from two drops of cationic chitosan and microparticles dispersed poly(styrene sulfonate)sodium salt solution, respectively, biocomponent filaments were fabricated, which demonstrated reversible humidity‐dependent shape change.

A similar approach was made with negatively charged TCNF from sugarcane bagasse and cationic chitosan.^[^ ^24^ ^]^ Two drops were placed next to each other on a Teflon surface generating the interface that could be picked up and drawn with a pair of tweezers, and the filament could be drawn as long as there was liquid in the drops (Figure 5e–g). The nanocellulose–chitosan composite filaments showed good mechanical properties with tensile strength and Young's modulus of 220 MPa and 22 GPa, respectively. Furthermore, it was argued that the drawing process induces alignment of the nanocellulose as well as of the chitosan. The fabricated filaments also showed the typical nervation/veining pattern on the surface.

The interfacial complexation strategy has also been applied to fabricate all‐cellulose filaments.^[^ ^25^ ^]^ Using cationic CNC as the positively charged component, three anionic celluloses were used; soluble sodium carboxymethyl cellulose, and negatively charged insoluble cellulosic nanoparticles in the form of TCNF and CNC. The use of two different oppositely charged nanocelluloses is referred to as interfacial nanoparticle complexation. These filaments also proved to be the strongest and had a tensile strength of 153 MPa and Young's modulus of 8.4 GPa. Also, for these filaments, the typical nervation/veining pattern could be found on the surface and by fanning out of wet filaments.

## 3 Improvements and Tuning of Filament Properties

Apart from the filament forming mechanisms present in different spinning processes, there are other means that can be used to modify filament properties, such as process parameters, chemical adjuvants and posttreatments such as stretching and twisting.

### 3.1 Improving Alignment Through Spinning Rates

The nanofibrillar alignment can be improved by the increase in spinning rate. In this perspective, spinning of nanocellulose suspension may have some similarities to the spinning of polymer solutions above the overlap concentration or of polymer melts. It has been shown that the increase in deformation rate (i.e., spinning rate) would enhance the orientation of polymer chains,^[^ ^26^ ^]^ which should also be the case for nanocellulose spinning. However, the factor that can influence the orientation of nanofibrils (CNF) goes beyond just the spinning rate along, it also depends on the stress involved during the process, which is a function of suspension viscosity or concentration. Perhaps the more reasonable consideration is through the input of specific work, which is the product of stress and strain (strain = the product of spinning rate and duration of spin line).^[^ ^27^, ^32^ ^]^ The specific work has been seen as the critical factor to produce oriented chains that lead to production of oriented nuclei in flow‐induced polymer crystallization during fiber spinning. The increase in specific work will no doubt also enhance the orientation of nanofibrils during spinning.

### 3.2 Crosslinking as a Means to Improve Filament Properties

**Figure 6:**
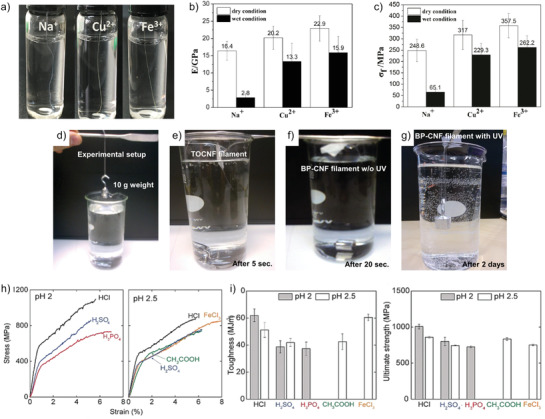
a–c) Improved mechanical properties by ionic crosslinking.^[^ ^28^ ^]^ a) Contrast photos of filaments swelling, b) tensile strength, and c) Young's modulus in dry and wet state. d–g) Illustration of improvement of wet‐strength by photo‐crosslinking.^[^ ^29^ ^]^ d) A 10 g weight is loaded on the filament, which is suspended from a beam followed by immersion in water. e) TCNF filament and weight after 5 s in water, f) BP‐CNF filament without UV treatment after 20 s in water, and g) BP‐CNF filament with UV treatment after 2 days. h–i) Tensile mechanical properties of CNF filaments.^[^ ^32^ ^]^ h) Representative stress–strain curves of fibers prepared at pH 2 and 2.5; i) toughness and h) ultimate strength, where error bars are 90% confidence intervals. a–c) Adapted with permission.^[^ ^28^ ^]^ Copyright 2017, American Chemical Society. d–g) Adapted with permission.^[^ ^29^ ^]^ Copyright 2017, American Chemical Society. h,i) Adapted with permission.^[^ ^32^ ^]^ Copyright 2019, Wiley‐VCH.

Enhanced interfacial (surface) interactions may improve mechanical performance in both dry and wet state.^[^ ^28^ ^]^ In one example study involving ionic crosslinking agents, after wet spinning filaments from bacterial cellulose nanofibers into acetone and drying, the filaments were immersed into solutions of CuSO_4_ and Fe_2_(SO_4_)_3_ for 24 h, followed by extensive washing and drying. Filaments were then tested dry and wet (**Figure** **6**a–c). The dry filaments had maximum tensile strength of 248.6 MPa and Young's modulus of 16.4 GPa, and after ion exchange the mechanical properties in wet conditions were still good with a maximum tensile strength and Young's modulus of 262.2 MPa and 15.9 GPa, respectively. Strain at fracture was clearly reduced after ion exchange, but was in principle unaffected by the dryness of the filaments.

As a means to improve wet‐strength it was shown that photo‐crosslinking can be utilized to produce filaments using wet spinning,^[^ ^29^ ^]^ where benzophenone‐activated wood‐pulp TCNF was used as the spinning dispersion.^[^ ^30^ ^]^ The dry filaments were UV‐treated and characterized with respect to mechanical properties in dry and wet states. The crosslinked filaments showed significantly lower apparent density (0.70 g cm^−3^) compared to pure TCNF‐filaments (1.22 g cm^−3^) that were used as reference. In dry state the crosslinked filaments showed inferior tensile strength and Young's modulus properties compared to the TCNF‐filaments, which probably can be attributed to the clearly lower density. When specific tensile strength is considered, no significant difference can be observed, and the crosslinked filaments showed slightly higher specific modulus. However, when comparing the performance between dry and wet filaments, the crosslinked fibers retain ≈81% of the dry strength, which is demonstrated in Figure 6d–g.

Physical and chemical crosslinking can also be used for improving filament properties, which has been shown by adding polyamide‐epichlorohydrin (PAE) to a jute TCNF dispersion.^[^ ^31^ ^]^ The dispersion was wet‐spun into an acetone coagulation bath and heat treated after drying. Apart from a clear relation between mechanical properties and spinning speed, it was shown that the crosslinking increases the tensile strength of 268.7 MPa and Young's modulus of 22.8 GPa for the pure‐CNF‐filaments, to 369.8 MPa and 28.9 GPa, respectively, for the crosslinked filament with no significant effect on strain at failure.

By using the flow‐focusing technique, the possibility to vary sheath flow composition was explored to investigate gel initiation (coagulation) mechanisms.^[^ ^32^ ^]^ A set of acids were applied as well as FeCl_3._ The latter promotes ionic crosslinking of nanofibrils giving strong hydrogel networks.^[^ ^33^ ^]^ Spinning was done using a double flow‐focusing device with a carboxymethylated wood‐pulp CNF dispersion as the core flow at 0.2–0.3 wt%. The first sheath flow was deionized water and the second sheath flow was varied between HCl, H_2_SO_4_, H_3_PO_4_, CH_3_COOH, and FeCl_3_. The three first acid‐coagulated filaments were spun at pH 2 and 2.5. The evaluation of mechanical performance (Figure 6h,i), showed that the filaments spun at low pH with HCl gave the best mechanical properties with a tensile strength of 1010 MPa, a Young's modulus of 57 GPa and a strain to failure of ≈6%. These favorable properties also resulted in a toughness of 62 MJ m^−3^. The use of FeCl_3_ reduced the strength and stiffness compared to HCl but improved strain to failure.

### 3.3 Increased CNF Alignment and Filament Performance by Stretching and Twisting

#### 3.3.1 Postspinning stretching

**Figure 7:**
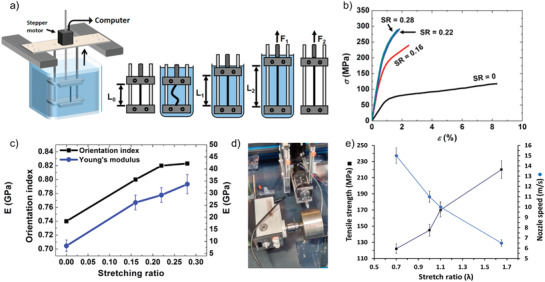
a–c) Stretching of filaments after drying.^[^ ^34^ ^]^ a) Schematic drawing for a computer‐controlled wet‐stretching device. b) Tensile strength properties of wet‐spun CNF‐filaments as a function of stretching ratio (SR). c) Variation of the orientation index the Young's modulus, as a function of the stretching ratio. d,e) Online stretching of dry‐spun filaments.^[^ ^36^ ^]^ d) Device for dry‐spinning on a rotating drum. e) Correlation between stretching ratio and tensile strength. a–c) Adapted with permission.^[^ ^34^ ^]^ Copyright 2014, American Chemical Society. d,e) Adapted with permission.^[^ ^36^ ^]^ Copyright 2016, Springer.

By stretching dried filaments after rehydration, the change of CNF alignment has been studied (**Figure** **7**a).^[^ ^34^ ^]^ This study also compared CNF‐based filaments to filaments made from chitin nanofibrils. The results showed that the stretching of a filament improved the tensile strength and Young's modulus (Figure 7b), where there was an almost linear relation between the two (Figure 7c). This study also showed that there is a relation between the stretching ratio and the alignment of the crystalline domains. It was suggested that the alignment through stretching is more effective than the alignment caused by elevated processing speed. Furthermore, the effects of entanglement were discussed where it was suggested that the nanofibril length coupled to the shear rate also have an impact. It was thought that the poststretching would homogenously affect the structure of the full filament, whereas stretching during coagulation might result in nonuniform alignment in the final filament as the mobility of CNF along the radial direction would vary. Finally, they noted that the properties of CNF‐based filaments were more affected by the formation of defects.

The results from varying filament spinning studies showed clear correlations between the nanofibril alignment and mechanical properties (i.e., strength and stiffness), whilst strain‐at‐break ratio always decreases with the increasing CNF alignment. In order to circumvent the negative effect of nanofibril alignment on the strain‐at‐break ratio, filaments were wet‐spun using wood‐pulp TCNF with CaCl_2_ as a coagulant to induce ionic crosslinking.^[^ ^35^ ^]^ The study showed that at the same spinning conditions (≈40 m min^−1^), unstretched filaments exhibited a Young's modulus of 14.5 GPa, strength of 235 MPa, and strain‐at‐break of 10.2%. With poststretching, filaments exhibited a Young's modulus of 21 GPa, strength of 383 MPa and strain‐at‐break of 7.1%. This indicated both filaments (unstretched and stretched) has similar toughness of ≈17 MJ m^−3^.

In a follow‐up investigation, a step‐wise process was used to fabricate filaments from a dispersion of 2 wt% wood‐pulp TCNF also with CaCl_2_ as the coagulation agent. As the first step, the effect of the shear rate during spinning was evaluated. Based on these results, the effect of predrying temperature, i.e., drying after coagulation, was subsequently explored. Finally, the optimal inner diameter of the needle was also identified. The optimal spinning condition was used as the starting point to produce filaments for stretching experiments at 95% RH. The results showed that 20% of stretching could produce filaments with strength of 543.1 MPa, stiffness of 37.5 MPa and strain‐at‐break ratio of 3.7%. Without stretching, filaments only exhibited the strength of 492.6 MPa, stiffness of 24.3 MPa and strain‐at‐break ratio of 11.8%. The observation clearly indicated that 20% of stretching increased the orientation index, from 0.77 to 0.86. In contrast, 10% of stretching increased the orientation index to 0.81 and stiffness by 30%, with no impact on the strength but reduced the strain‐at‐break ratio to half.

#### 3.3.2 Online Stretching

Using a dry spinning approach, it was possible to study the effect of several processing parameters during online (in situ) stretching of wood‐based TCNF‐filaments on their properties.^[^ ^36^ ^]^ In this study, the dispersion was extruded onto a rotating drum, where the stretch of the spun filament could be controlled by varying the extrusion speed of the filament and the speed of the rotating drum. This set‐up not only allowed the online control of filament stretching up to 70%, but also allowed processing at high speeds up to 660 m min^−1^ (11 m s^−1^). The interactions between the spun filament and the surface of the rotating drum (polyethylene (PE) coated with aluminum) could be further controlled by the coating of sunflower oil while the drum was rotating (an estimated coating weight was 1–3 g m^−2^), resulting in a layer thickness between 1 and 3 μm. The oil coating was shown to be necessary for fabricating a filament with a uniform cross‐section. The results further showed a strong correlation between stretching and tensile strength, where 70% stretch was able to yield the strength of 220 MPa (Figure 7d,e).

By using a similar set‐up, the effect of heating during drying of the filaments based on nonmodified CNF from wood‐pulp was investigated. The starting material was prepared by a microgrinder where the effect of nanosuspension composition was also evaluated.^[^ ^37^ ^]^ Heating of the filament was achieved using a hot‐air gun, where it was found that the drying temperature only had a minor effect on the resulting filament, where the grinding treatment time exhibited a more profound impact. This process was further adopted for the investigation of the effect of surface upon which filaments are formed during wet spinning.^[^ ^38^ ^]^

#### 3.3.3 Effects of Postspinning Twisting

As a different but effective route to fabricate strong filaments, sheets of bacterial nanocellulose could be twisted.^[^ ^39^ ^]^ Apart from developing the hierarchical structure, the twisting process also promoted enhanced dewatering. Twisting was performed after the initial stretching of the filament, where stretching was found to have a significant effect on the filament properties. In this study, samples were cut into 7 × 70 mm sheets that were stretched by 30% before twisting. The resulting filament exhibited very high strength of 825 MPa, Young's modulus of 65.7 GPa and a strain‐at‐break ratio of 2.5%. In contrast, at no or low stretching (e.g., 10%), the strength, stiffness and strain‐at‐break ratio were 130 MPa, 4.3 GPa, and 20.6%, respectively. It was interesting to find that at the 20% stretch, the stiffness increased to 17.4 GPa with a strain‐at‐break ratio reduced to 5.2%, but the tensile strength increased only slightly to 194.9 MPa.

The twisting approach was also applied to wet‐spun filaments using blends of alginate and bacterial nanocellulose (up to 50 wt%),^[^ ^40^ ^]^ where the filaments were first formed in a CaCl_2_ coagulation bath. It was found that both strain‐at‐break ratio and toughness increased linearly with the number of filaments used in the twisted configurations, whereas the strength showed a maximum value with only 4 filaments involved in the twist. For bundles of parallel filaments, no significant effects were found regarding the number of filaments used. The maximum strength obtained was 535.4 MPa for a twisted rope consisting of 4 filaments, having a strain‐at‐break ratio of 12.2%, and toughness of 38.3 MJ m^−3^.

### 3.4 Structural Scales and Functionalization of Nanocellulose Filaments

**Figure 8:**
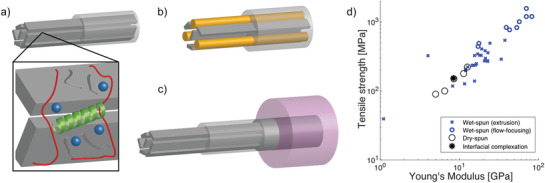
Routes for functionalization. a) Nanoscale functionalization where added components are of the same dimension as the diameter of the nanofibrils, b) mesoscale functionalization where added components are of the same dimension as the nanofibril length, and c) macroscale functionalization where the added components are of the same dimension as the diameter of the filament. d) An overview of the tensile strength and Young's modulus of nanocellulosic filaments from published results, a corresponding table can be found in the Supporting Information.

Functionalization of CNF‐filaments can be achieved in many ways and is here structured into three main routes (**Figure** **8**a–c). The first route, nanoscale functionalization, represent concepts where functionalization is achieved by adding components where the maximum effective dimension is similar to, or smaller than the diameter of the nanofibrils (Figure 8a). Components can be mixed into the CNF dispersion, or transported into the filament by diffusion during or after the spinning process. Thus, the components are so small that they can be transported through the nanofibrillar network. In the second route, referred to as mesoscale functionalization, components have one or more dimensions that are similar to the length of the nanofibrils, i.e., clearly larger than the nanofibril diameter (Figure 8b). The nanofibril network significantly hinders components moving around; thus, they most probably need to be mixed into the dispersion. For the third route, components either have dimensions larger than the length of the nanofibril, or the dimensions of the final structures have dimensions similar to the filament diameter (Figure 8c). Thus, the latter includes the case when a filament is coated or given a core–shell structure, where the coated surface or shell represents a coherent entity with a dimension of the filament diameter.

To some extent, all published results that aim at fabricating filaments with improved mechanical properties, wet or dry, have used various ions or polymeric crosslinkers. Thus, this represents nanoscale functionalization enhancing existing properties of the macroscopic filament by mediating nanoscale interactions of the nanofibrils. In Figure 8d, a selected set of results on mechanical performance are plotted as tensile strength versus Young's modulus. This can also be found as a table in the supporting information. Apart from boosting existing properties (functionality), it is also possible to add new functionalities to filaments by introducing components with other properties, aiming at a “superposition” of the properties from both components, i.e., hybrid materials.^[^ ^41^ ^]^

#### 3.4.1 Functionalization for Biocompatibility and Bioactivity

One possible advanced use of nanocellulose filaments is for biomedical purposes.^[^ ^42^ ^]^ Using a commercial CNF, it was demonstrated that it is possible to fabricate human stem cell decorated nanocellulose filaments for biomedical applications.^[^ ^43^ ^]^ After drying in room conditions filaments were immersed in a solution with glutaraldehyde and zinc nitrate for nanoscale functionalization, and subsequently heat treated to induce chemical crosslinking between nanofibrils. The crosslinked filaments soaked in water was shown to retain about 50% of their strength at 50%RH (calculated using the dry cross‐sectional area). It was extensively show that the filaments support attachment of human stem cells without causing any deterioration and that they preserved bioactivity without inducing any toxicity.

As another example of nanoscale functionalization, UV‐crosslinked, wet‐spun wood‐pulp TCNF filaments, discussed above, represents a platform for development of advanced functional materials, which was demonstrated through biomolecular detection or affinity adsorption.^[^ ^29^ ^]^ The affinity binding was used to conjugate antihuman hemoglobin (anti‐Hb) antibodies onto residual surface carboxyl groups of the spun filaments, rendering them bifunctional (wet strength and bioactivity).

Apart from the improvement regarding mechanical properties, the addition of recombinant spider silk proteins provided additional nanoscale functionality for biomedical applications.^[^ ^16^ ^]^ In one study, two types of silk fusion proteins: Z‐silk and FN‐silk were used. In the case of Z‐silk, the silk protein was fused to an affinity domain Z that provided the ability to selectively bind immunoglobulin G (IgG).^[^ ^44^ ^]^ In FN‐silk, the enhanced functionalization was directed toward the improvement of cell‐binding capability.^[^ ^45^ ^]^ The fabricated filaments showed a surface bioactivity using 90% CNF that is similar to materials based on pure silk fusion proteins.

Nanocellulose processed from oil palm empty fruit bunches (OPEFBs) was used to fabricate filaments, which were compared to neat poly(vinyl alcohol) (PVA)‐filaments and PVA‐bacterial nanocellulose filaments as control.^[^ ^46^ ^]^ The filaments were wet‐spun and subsequently coagulated in acetone. For these hybrid filaments, nanocelluloses were added in the range 10–30 wt%, and mechanical tests were performed in dry and wet states. The results also showed that cell viability increased slightly when nanocellulose was added, i.e., in principle the nanocellulose was used to provide nanoscale functionalization of PVA filaments. The cell metabolic activity was evaluated using an MTT assays test. The resulting filaments showed at least 50% cell viability after two weeks, whereas the pure nanocellulose filaments did not show any between 7 and 14 days.

#### 3.4.2 Functionalization for Conductivity and Sensor Applications

**Figure 9:**
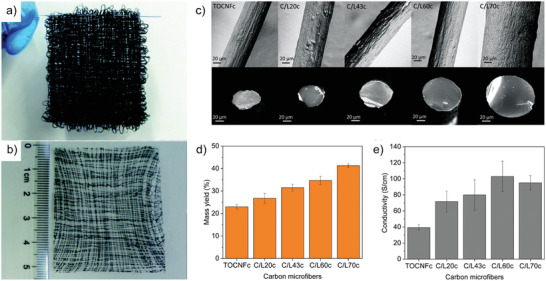
a) Wet and b) dry CNF/SWCNT hybrid nonwoven filament mat.^[^ ^47^ ^]^ c–e) Carbonization of CNF‐filaments.^[^ ^53^ ^]^ c) CNF surface morphologies and cross‐section at break from precursors with different lignin content. d) Mass yield of TCNF and lignin bicomponent filaments after carbonization at 900 °C for 60 min. e) Electrical conductivity of CMFs obtained from precursor filaments with a given lignin content. a,b) Adapted with permission.^[^ ^47^ ^]^ Copyright 2014, The Royal Society of Chemistry. c–e) Adapted with permission.^[^ ^53^ ^]^ Copyright 2019, American Chemical Society.

The possibility to use CNF‐based filaments as the starting material for electrical devices was demonstrated through mesoscale functionalization by adding single‐walled carbon nanotubes (SWCNTs) to the CNF dispersion.^[^ ^47^ ^]^ In this demonstration, a dispersion was prepared by adding 0.69 g nonmodified SWCNT to 100 g of 0.48 wt% CNF suspension to make a wearable supercapacitor. With the syringe‐based spinning technique, filament mats were fabricated by extruding the mixture dope into ethanol. The resulting filament after air drying exhibited an average diameter of about 50 μm (**Figure** **9**a,b). It was found that the CNF dispersion prevented aggregation of SWCNT as well as improved the re‐swelling properties in the presence of aqueous electrolytes. The mats were shown to have good electrochemical properties, electrochemical stability and damage reliability. The good electrochemical performance was attributed to an efficient ion diffusion process in the mats, provided by the macro‐ and nanoporous structures as well as high porosity.

The addition of SWCNTs at higher concentrations has also been pursued using the flow‐focusing spinning technique.^[^ ^48^ ^]^ Filaments were fabricated at different levels of SWCNT addition,and it was shown that the low‐concentration CNF is an excellent dispersing agent for SWCNT. The filaments were spun from a 0.3 wt% dispersion, where the dry filaments consisted of 43 wt% of SWCNT. The strength and Young's modulus of the spun filaments was 220 MPa and 14 GPa, respectively, and the measured conductivity was above 200 S cm^−1^ and current densities reached 1400 A cm^−2^. A similar approach was also made using wet spinning with a coagulation bath with FeCl_3_ and HCl. Conductivity reaching a maximum of about 100 S cm^−1^, demonstrating use the filaments as a strain sensor for measuring mass variation and muscular movement.^[^ ^49^ ^]^

CNT was also mixed with TCNF from *Halocynthia roretzi* (sea pineapple) and wet‐spun to produce chemical‐sensing filaments with good mechanical properties (tensile strength of up to 240 MPa, Young's modulus up to 19 GPa), electrical conductivity, and highly porous surfaces.^[^ ^50^ ^]^ The fabricated filaments were shown to exhibit a clear gas‐sensing (NO_2_) performance with high selectivity and sensitivity. It was also shown that the filaments could endure complex and harsh distortions.

Instead of mesoscale functionalization using carbon nanotubes, it has also been shown that filaments can be wet‐spun from a 50/50 mixture of graphene oxide (GO) and CNF into ethanol.^[^ ^51^ ^]^ Final filament diameter was varied in the range of 10–100 μm by changing needle diameter and the dried filaments were carbonized and evaluated with respect to conductivity, which reached 649 S cm^−1^. This was attributed to the carbonization of well‐aligned CNF–GO, and pure CNF and GO filaments were used as references. The CNF–GO also gives rise to increased mechanical performance.^[^ ^52^ ^]^

A blend of wood‐derived lignin and wood‐pulp TCNF can also be used to fabricate precursor filaments for carbonization.^[^ ^53^ ^]^ Using a wet spinning set‐up with CaCl_2_ as a coagulation agent, the lignin content of the spinning dope was varied from 0 to 70 wt%. The resulting filaments showed typical mechanical performance for the case of pure TCNF, where decreasing performance was found with increasing lignin content. This study indicated that there is no need for stabilization prior to carbonization. In the images of the cross‐sections of carbonized filaments, no aggregation or fusion of lignin could be detected. Perhaps, this is because the CNF protected lignin from fusion during heating. It was seen that the mass yield after carbonization increased with increasing lignin content (Figure 9c–e), as did the conductivity.

It has also been shown that it is possible to fabricate color‐tunable luminescent filaments from TCNF processed from membranes obtained by cultivating *gluconacetobacterxylinus*.^[^ ^54^ ^]^ The nanofibrils were nanoscale functionalized with Cadmium Telluride quantum dots (CdTe‐QDs), which were grown in situ on the individual nanofibrils. The QDs were evenly distributed on the nanofibrils without aggregation. The fabricated filaments were luminescent with a fluorescence intensity that depends on pH and glucose content and can thus be used as biosensor materials.

By wet spinning of wood‐pulp based carboxymethylated CNF‐filaments into a coagulation bath with HCl and aluminum‐modified silica nanoparticles (SNP), a concept for improving flame retardancy was demonstrated, which was attributed to the char forming and heat capacity capabilities of the silica nanoparticles.^[^ ^55^ ^]^ Despite significant shrinkage during drying, the spinning was performed at 0.75 wt% concentration, the fabricated filaments showed a well‐defined core–shell structure having a cellulosic core and a shell of homogeneously distributed silica nanoparticles, thus an example of macro‐scale functionalization. It was suggested that this was due to rapid interfacial complexation between the negatively charged CNF and the aluminum‐modified SNP. It was also shown that addition of SNP reduced the tenacity (tensile strength), but preserved the Young's modulus of the fibers.

As another example of macroscale functionalization, hydrophobized using chemical vapor deposition of trichloromethylsilane (TC) or dimethyldichlorosilane (DC) was used to improve wet strength of wet spun wood‐pulp TCNF filaments.^[^ ^56^ ^]^ The DC‐modified filaments presented a rather homogeneous surface coverage, characterized by a smooth and soft poly(dimethylsiloxane)‐like coating. In contrast, amphiphobic TC‐modified filaments included hairy structures self‐assembled on their surfaces. Both types of filaments presented improved stability in water but retained a similar level of moisture sorption compared to the unmodified precursors. Compared to the nontreated filaments, the wet tensile strength of DC filaments was three times compared to unmodified filaments. The results provide a route for filaments hydrophobization that could function in applications demanding water repellence combined with breathability, such as wearables for outdoor use.

## 4 Challenges Related to the Spinning Process and Successful Assembly of Nanocellulose Filaments

In the previous sections, reported conditions for spinning and various nanocellulose fiber concepts have been discussed. To make CNF‐spinning a viable engineering process for fabrication of high‐performance fibers, the current knowledge regarding the mechanisms that control the structure formation at multiscale during filament formation needs to be developed further. Many of the current publications does not contain sufficient information to readily reveal the underlying mechanisms that control the nanoscale assembly process(es). To address this, we will try to elucidate how the nanoscale assembly can be: 1) understood based on fundamental principles of particle motion in flows, 2) characterized with novel in situ characterization techniques to capture dynamics and structural changes during flow, and 3) controlled and improved based on theoretical and numerical models.

Being a rather new scientific field, general arguments with respect to nanocellulose rheology, nanofibril mobility, and fibrillar interactions can take advantage of the knowledge base generated from two industrial material manufacturing fields: polymer processing and papermaking. In our opinion, confusion often arises when nanocellulose dispersions are described as polymer solutions. It is equally confusing to compare the nanocellulose dispersions to wood‐pulp fiber slurries used in papermaking. Due to the very thin cross‐sections of cellulose nanofibers, it is also likely that colloidal theories break down.^[^ ^57^ ^]^

### 4.1 Mechanisms that Control Nanocellulose Dispersions During Spinning

#### 4.1.1 Sizes, Shapes, and Lengthscales

**Figure 10:**
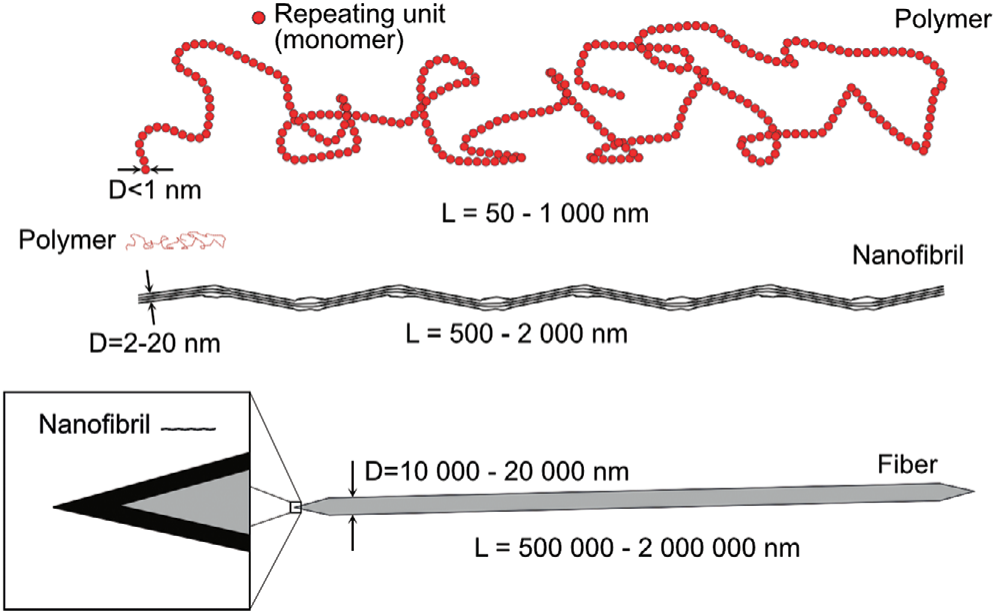
Illustration of the differences in length scales between polymers, cellulose nanofibers, and cellulose fibers. A polymer is a long chain of repeating molecular sized units (monomers) attached through covalent bonds. A nanofibril consists of 10–100 cellulose polymer chains in a crystalline formation through hydrogen bonding, with varying degree of crystallinity along the length and width. A cellulose fiber typically consists of more than 10^10^ nanofibrils. The typical width of a polymer is less than a nanometer, while a nanofibril can be up to tenths of nanometers and a fiber exceeds a micrometer in thickness. This difference in scales is crucial in understanding the influence of thermal motion and properties of the individual elements when processing them into useful materials.

The differences in length scales between polymers, nanofibrils and cellulose fibers are schematically illustrated in **Figure** **10**.

##### Polymers

A single polymer chain consists of repeating units, where each repeating unit is of the size of 0.2–1 nm. The chain length is given by the number of the repeating unit, i.e., degree of polymerization (DP), which can be considered as a measure of the aspect ratio (often over 1000).^[^ ^58^ ^]^ The polymer chain can be linear or can have branches/side chains, which affects the packing density, e.g., high‐density polyethylene (HDPE) and low‐density polyethylene (LDPE) chains. The cellulosic polymer chain does not possess any side chains, and forms highly crystalline arrangements facilitated by strong inter‐ and intrachain hydrogen bonding.^[^ ^59^ ^]^

##### Cellulose Fibers

Macroscopic cellulose fibers originate from plants such as trees,^[^ ^60^ ^]^ where high aspect‐ratio cells are typically referred to as “cellulose fibers.” They are 10–20 μm thick with a length of 1–2 mm. During pulping, the hemicelluloses and lignin surrounding the cellulose‐based cell are chemically degraded and removed, thus liberating the cellulose fibers to form so‐called pulp suspensions used in, e.g., papermaking.

##### Cellulose Nanofibers

Cellulose nanofibers consists of only a few cellulose polymer chains packed closely and bonded with van der Waals forces and hydrogen bonds.^[^ ^59^ ^]^ They are formed in the cellulose synthase (rosette) complexes in the secondary cell walls of plants, each consisting of six globular proteins where the cellulose polymer chains are synthesized,^[^ ^61^ ^]^ which in turn forms a close‐packed assembly, i.e., elementary nanofibril or the building block of nanocellulose. The synthesis is, however, not perfect and the nanofibril can consist of both domains of highly crystalline arrangement and less ordered structure (*para*‐crystalline with defects or amorphous).^[^ ^62^ ^]^ The crystalline arrangement gives the nanofibril mechanical rigidity and thus more particle‐like properties compared to individual polymer chains. Through different chemical, enzymatic and mechanical processes, the cellulose nanofibers can be liberated, either as elementary nanofibrils or as bundles of elementary nanofibrils. Our focus will be here of CNF extracted through high oxidation and homogenization, resulting in typical cross‐section dimensions of 2–10 nm and lengths up to a micrometer. However, the discussion will also be highly relevant both for cellulose nanocrystals (CNC; lengths 100–250 nm, widths 5–70 nm) and bacterial nanocellulose (BNC; widths 20–100 nm and forming fibrillar networks rather than individual fibrils).^[^ ^63^ ^]^

#### 4.1.2 Thermal Motion and Conformation (or Deformation) of Single Particles

There are notable differences between polymers, nanofibrils and cellulose fibers with respect to effects of thermal motion and corresponding conformational (deformational) changes.

##### Polymers

Thermal motion is crucial to the shape of the single polymer chain. For example, in polyolefin, the bond angle between two adjacent carbons is fixed, but the atoms can rotate around the single bond, which can lead to different conformations of the chain.^[^ ^64^ ^]^ The bending stiffness of the polymer chain is often characterized using the persistence length, which in principle is a measure of how long the portion of the chain can retain their shape under thermal fluctuations. For a typical polymer (e.g., polyolefin) at room temperature, the persistence length is much lower than the actual contour length, meaning that thermal motion has a strong influence on the polymer shape and stiffness. The typical time scales for major conformational changes in an aqueous solution will be on the order of nanoseconds (<10^−9^ s).^[^ ^65^ ^]^

##### Cellulose Fibers

The macroscopic cellulose fiber from, e.g., wood can be well described as a semiflexible and essentially rod‐like particle. In a wood‐pulp suspension flow, the particles can be bent by hydrodynamic forces during the process, but the deformation (bending) is typically elastic, and they tend to relax back to their original shape. Thermal motion is negligible and the time scale for to rotate a significant angle for cellulose fiber in water would be several months. Thus, buoyancy forces are much more important with respect to the motion of cellulose fibers.

##### Cellulose Nanofibers

The mechanical properties of the nanofibril is still the focus of extensive research and therefore less known. The crystalline arrangement of cellulose chains in nanofibrils and strong intermolecular hydrogen bonding restricts the conformational changes due to thermal motion at room temperature. It is also reasonable to assume that the bending stiffness is lower in noncrystalline domains than that in crystalline domains, thus rendering the nanofibril semiflexible, similarly to the cellulose fiber. Still, the average persistence length of the nanofibrils is most probably somewhere on the same scale as the actual length or larger, and the flexibility/stiffness could also be differing depending on the raw material source and treatment methods to liberate the nanofibrils. Given the small size of the nanofibril, their orientation in an aqueous dispersion is highly affected by thermal motion of surrounding solvent molecules. The typical time scale for thermal motion to rotate an elementary nanofibril will be on the order of sub‐milliseconds (<10^−3^ s).

#### 4.1.3 Particle–Particle Interactions in Dilute Dispersions (Single Particles)

##### Polymers

When one polymer chain is approaching another polymer chain, the counteracting forces mainly consist of electrostatic repulsion of charged groups if they exist or otherwise just direct contact.^[^ ^67^ ^]^ However, if a polymer chain pulls away from another chain or move in parallel to another chain, the counteracting forces are mainly due to secondary van der Waals forces or hydrogen bonds. A polymer chain can also be crosslinked by adding a compound that can bind to the side groups on two nearby polymer chains. This primary bond will of course also act as a strong counteracting force to receding polymer chains.

##### Cellulose Fibers

For macroscopic cellulose fibers, the significant forces occurring at a fiber–fiber contact point relates to the normal force and resulting friction force between the nonsmooth fiber surfaces. Of course, electrostatic and van der Waals forces between the fibers will exist when in direct contact, but they are several orders of magnitude weaker than mechanistic forces. Thus, in cellulose fiber suspensions,^[^ ^68^ ^]^ the frictional forces become the dominating factor if the fibers are in direct contact, which is the case when so‐called fiber flocks are formed.

##### Cellulose Nanofibers

In terms of particle–particle interactions, the nanofibrils are often regarded as a colloid with interactions being described with Derjaguin–Landau–Vervey–Overbeek (DLVO) theory. To be able to have a stable dispersion, charged side groups on the nanofibrils can form strong electrostatic repulsion to prevent aggregation.^[^ ^14^ ^]^ If the repulsive force is reduced, typically through the addition of salt or acid, the nanofibrils can come close enough due to the attractive intermolecular forces (e.g., van der Waals) to form secondary fibril–fibril bonds. The forces can be modelled through attractive and repulsive continuous potential fields surrounding the particles. There are however reasons to argue why DLVO‐theory is not appropriate for nanofibrils.^[^ ^57^ ^]^ The reason is that the cross‐section of a nanofibril in the case of CNF is somewhere between 1 and 5 nm, which is close to the size range of hydrated ions in the solvent.^[^ ^33^ ^]^ One of the crucial assumptions in DLVO‐theory is that solvated ions are negligibly small compared to the nanoparticles, which of course would not be the case with respect to the thin nanofibrils. For this reason, the cellulose nanofiber probably has more in common with charged polymer chains. Compared to other forces on the nanofibril particle, these interactions are however weaker than those in polymer chains as the surface area of a nanofibril is smaller. Still, the actual particle‐particle interactions between the nanofibrils are less known and is also an active research field, where novel experiments and molecular dynamics simulations probably will shed some light in the upcoming years.

#### 4.1.4 Particle–Particle Interactions in Semidilute or Concentrated Dispersions (Networks)

##### Polymers

A polymer can be dispersed in a solution depending on its solubility in the solvent, which is determined by the molecular interactions between polymer and solvent. Precipitations (aggregation) of interconnected polymer chains in an initially stable solution can be formed by: 1) increasing concentration (more polymer chains in the solution means more bonds), 2) increasing the chain length through DP (a higher aspect ratio means a more entangled network), 3) decreasing the length of side chains (polymers can pack more closely, which leads to more bonds), 4) addition of crosslinkers (compounds that cause stronger intermolecular bonding), 5) dipole strengthening (changing side‐groups on the polymer can cause stronger secondary bonds), 6) reducing temperature (lower thermal motion means less dissociation), and 7) phase inversion by solvent/nonsolvent exchange (changing the solvent affects the solubility of the polymers). It is worth mentioning that polymers usually can be processed without any solvent, as their internal thermal motion can make them transition from a solid to a viscoelastic liquid above the glass‐transition temperature.

##### Cellulose Fibers

For a suspension of macroscopic cellulose fibers, the effect of thermal motion is negligible. However, the viscosity of the solvent will affect the role of buoyancy forces and thus the rates of sedimentation, deposition, and resuspension, as well as laminar/turbulent transitions (thus indirectly influencing the fiber mixing in flow). The fibers can form flocks, which are a result of the fiber entanglement and does not represent any significant local increase in concentration, i.e., aggregation. The mechanisms behind this entanglement is the semi‐flexibility of the fibers and the friction at fiber–fiber contact points. Since the fibers are elastic, they can be bent into an entangled network where reptation is hindered by fiber–fiber friction, thus storing elastic energy. The propensity to form fiber flocks is mainly controlled by the so‐called crowding factor^[^ ^69^ ^]^ and friction at fiber–fiber contact points.^[^ ^70^ ^]^ The crowding factor *N* is defined as

(1) $$N=\frac{2}{3}\phi {\left(\frac{l}{d}\right)}^{2}=\frac{2}{3}\phi A^{2}$$

where ϕ is the concentration of the system, and the aspect ratio (*A*) equals to *l*/*d*, where *l* and *d* are length and diameter of the fibers, respectively. In principle, the crowding factor defines how many fibers can be found in the volume of a sphere having the same diameter as the fiber length. Assuming an isotropic fiber orientation distribution with rods, the number of fiber–fiber contacts can be estimated as

(2) $${\bar{n}}_{\text{c}}=\frac{3}{A}\;N$$

**Figure 11:**
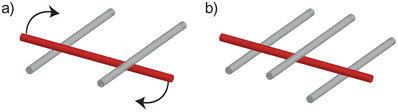
Illustration of the importance of number of fiber contacts for percolation of a 3D network of fibers. a) With 2 contact points a fiber still has some mobility in its rotation even though translation can be limited. b) With 3 contact points both rotation and translation can be suppressed.

The percolation in a 3D network requires ${\bar{n}}_{\text{c}}\geq 2$. In order to entrap a fiber in a network at least three contact points are needed as illustrated in **Figure** **11**. Since fibers are semiflexible and can be bent into a network structure, they can form flocks at a much lower concentration than predicted by ${\bar{n}}_{\text{c}}$.^[^ ^70^ ^]^ Just like polymers becoming more entangled when increasing concentration or DP, flocculation of fibers in a suspension will increase when the crowding factor increases, either due to an increase in particle concentration and/or aspect ratio, as both would increase the average number of contacts between fibers. Increased fiber–fiber interactions also strongly affects rheology and individual fiber mobility.^[^ ^71^ ^]^

##### Cellulose Nanofibers

To keep nanofibrils dispersed in a solvent, they need to be charged to induce electrostatic repulsion and prevent secondary bonding forming attractive intermolecular forces between the nanofibrils. For CNF this is most often done by adding negatively charged carboxylate (COO^−^) side groups to the nanofibril.^[^ ^72^, ^73^ ^]^ The nanofibrils can form a volume arrested state (VAS) either by: 1) increasing concentration or 2) reducing the electrostatic interactions between nanofibrils.^[^ ^74^ ^]^ In the first case, the threshold concentration is aspect ratio dependent, just like percolation of a macroscopic fiber network, i.e., that nanofibril mobility is hindered by the amount of average contact points. The interaction between nanofibrils is still repulsive leading to a reversible VAS called a “glass,” where nanofibrils will be redispersed upon dilution and mixing. In the second case, the VAS is formed by decreasing the electrostatic interactions between nanofibrils through addition of salts or acids, so that they can come close enough for secondary bonds to form. At lower pH level, there will be an excess of hydrogen (H^+^) ions that will bind to the carboxylate groups, thus neutralizing the electrostatic charge of the nanofibrils. In the presence of monovalent salts, positive ions will screen the carboxylate groups and leading to effectively less electrostatic repulsion between the nanofibrils. The addition of multivalent ions might even act as electrostatic (or ionic) crosslinkers between carboxylate groups. The interaction between nanofibrils in attractive in this case, leading to an irreversible VAS called a “gel.”

All three systems undergo some form of transition from a mobile dispersion to an entangled network through the increase of aspect ratio (or DP for polymers) and concentration. The key point is that even though the polymer chain is more similar to the cellulose nanofiber in terms of lengths and dimensions, the high persistence length and semiflexibility of the nanofibril makes the nanofibrillar network more resembling the macroscopic cellulose fiber network. Additionally, just like cellulose fibers, cellulose nanofibers are not affected by temperature in the absence of water, making it fundamentally different than polymers. Although there might be weak thermal fluctuations inside nanofibrils, the shapes will remain intact and they will form a dry fibrillar network yielding a nanopaper/nanomembrane. Extrapolations can provide us with values of a glass‐transition temperature of around 250 °C, respectively for CNF materials. However, these temperatures are normally far higher than the degradation temperature of nanocellulose.^[^ ^75^ ^]^

#### 4.1.5 Dispersions in Shear Flows

**Figure 12:**
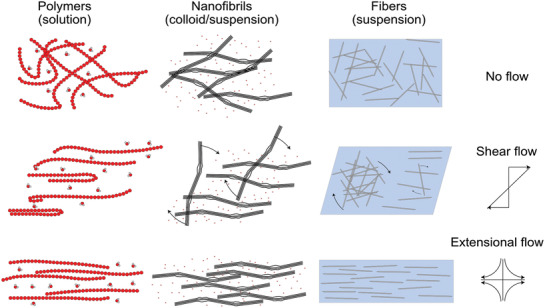
Illustration of how semidilute dispersions of polymer chains (atomic scale), nanofibrils (nanoscale) and fibers (microscale) behave in different flow fields. Shearing a polymer solution, leads to stretching and folding as well as disentanglement of the polymers. Shearing a fiber suspension typically leads to flock‐formation due to the semiflexibility of fibers and the vorticity of the shear flow. A dispersion of colloidal nanofibrils also have an inherent stiffness due to their crystallinity and is therefore also likely to behave like the fiber suspension, although being much more susceptive to Brownian motion of solvent molecules. In extensional flow, if hydrodynamic forces are stronger than Brownian motion, all systems will be purely aligned.

The behavior of polymers, nanofibrils and macroscale fiber dispersions in a shear flow are illustrated in **Figure** **12**, where the major features are a result of their differences as outlined in the previous sections.

##### Polymers

In a shear flow, dispersed polymer chains can disentangle due to hydrodynamic forces and thermal motion, causing secondary bonds to break.^[^ ^76^ ^]^ If the concentration is very high, cumulative secondary bonds will be stronger and require more work to deform the entangled polymer network, which is typically seen in increasing bulk shear viscosity. At higher shear rates, the rate of disentanglement becomes higher, leading to a decrease in viscosity, i.e., a shear thinning of the polymer solution. Due to bond stretching of polymer chains in the entangled polymeric network, the solution can also present viscoelastic properties depending on the concentration.

##### Cellulose Fibers

A stiff cellulose fiber in a shear flow will perform an intermittent rotational motion, but still can be aligned along the flow direction due to elongational forces.^[^ ^77^ ^]^ Due to rotation, the fiber will span a volume equivalent to a sphere having the diameter of the fiber length. If the equivalent spheres of the adjacent fibers can overlap, i.e., when the crowding factor *N* > 1, there will be an increase in fiber–fiber interactions, resulting in local flock formation.^[^ ^69^, ^70^ ^]^ The fiber‐fiber interactions also lead to an increasing apparent viscosity of the suspension, depending on the crowding factor. As flocks are formed, individual fibers will not align as the flock will rotates and stretches with the local vorticity. At low shear stress the fiber suspension will behave like an elastic solid in a strong interconnected network. Above a certain yield stress, the network is ruptured by the deformation and the suspension will behave like a shear thinning liquid.

##### Cellulose Nanofibers

The behavior of nanofibril dispersions in a shear flow is an interesting research topic that is still under development. Rheology has shown that the nanofibril dispersion is shear thinning, which is to be expected given the close relationship with both fiber suspensions and polymer solutions. Additionally, the nanofibril dispersions are usually thixotropic,^[^ ^72^, ^78^ ^]^ where the viscosity is time‐dependent with constant shear rate due to reversible structural changes.^[^ ^76^ ^]^

However, even for a stable nanofibril dispersion there are similarities to the mechanistic behavior of cellulose fiber suspensions. In a recent study,^[^ ^80^ ^]^ the crowding factor was used as a means to interpret rheological results. It was varied as a function of aspect ratio and concentration, and the effect of surface charge density (380–1360 μmol g^−1^) was evaluated. It was shown that the critical networking point of the dispersion, as determined by rheological measurements, was consistent with a so‐called gel crowding factor. Specifically, an anomalous rheological behavior was discussed that seemed to appear near the overlap concentration. The results were hypothesized to show the need to control flocculation and entanglement, which was used to understand favorable results obtained in earlier work.^[^ ^17^ ^]^ Recent results has further demonstrated how the collective motion of semidilute nanofibrils in confined shear flow aid alignment at low shear rates.^[^ ^81^ ^]^

These studies indicate that nanofibrils are semiflexible, resembling macroscopic cellulose fibers rather than the behavior of polymers in shear from a mechanistic perspective. The important difference the effect of rotary thermal diffusion, which will force the system toward isotropy.

#### 4.1.6 Dispersions in Extensional Flows

##### Polymers

Extensional flows will cause polymer chains to disentangle rapidly and stretch out (but not fold).^[^ ^76^ ^]^ The extensional deformation also leads to a decrease of lateral distances between aligned polymer chains, inducing orientation induced crystallization. Higher strain and speed of the extensional flow during spinning of polymer solutions and melts usually lead to high degree of chain orientation.

##### Cellulose Fibers

The extensional deformation of a fiber suspension can lead to a high degree of fiber orientation without any induced rotation. Any initial flocks ruptures given a high extension rate, and for the case of a flocculated fiber suspension, an extensional flow will also reduce the connectivity between individual flocks since the network strength of the flock is higher compared to the flock–flock connectivity strength.

##### Cellulose Nanofibers

The extensional deformation will also cause the nanofibrils to align, given high enough extension rate to overcome thermal rotary diffusion. However, the electrostatic repulsion is probably still strong enough for the nanofibrils not to come close enough to form stable secondary bonds in the aligned state. Still, it has been demonstrated that the rotary diffusion of semidilute nanofibrils could be dramatically reduced when subject to extensional flow.^[^ ^82^ ^]^

### 4.2 Routes for Controlling Nanoscale Alignment and Assembly During Spinning

The spinning techniques used for spinning man‐made fibers from polymer solutions or melts makes use of the transition between different conformations using shear and extensional flow‐fields. However, nanofibril dispersions should, as discussed above, be seen as a system between cellulose fiber suspensions and polymer solutions. The polymer‐like behavior is evident in the ability to form precipitations with the addition of counter ions (shielding the charged surface of cellulose nanofibers), demonstrating clearly the presence of van der Waals forces. The fiber‐like behavior is demonstrated clearly in the nonsensitivity of particle shape and bonds to thermal motion. Furthermore, the nanofibrillar system show clear similarities to the polymer solution at rest (where Brownian motion is dominating the dynamics), while behaving more like fiber suspensions during flow (when hydrodynamics prevail over Brownian motion). The dispersion of nanofibrils cannot be treated as a traditional colloid either.^[^ ^57^ ^]^ Thus, the dispersion of cellulose nanofibers should really be regarded as a system of its own, where a significant amount of interactions, structure, property, and process relations are still not well understood. However, it is possible to identify mechanisms that should be considered for the successful spinning of cellulose nanofibers into filaments.

#### 4.2.1 Flow Fields as a Means to Promote Alignment

**Figure 13:**
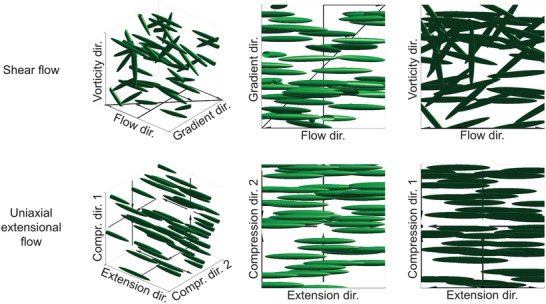
Illustration of the collective alignment of initially randomly oriented spheroidal particles of aspect ratio 10 according to the theory by Jeffery.^[^ ^77^ ^]^ In a shear flow, the spheroids are intermittently tumbling, but spending most of the rotational period with a projected alignment in the flow direction. This results in a very high perceived alignment when observing the system in the flow‐gradient plane, even though the 3D orientation distribution is much more isotropic. In a uniaxial extensional flow, the particles will immediately align in the stretching direction. Corresponding videos are provided as Supporting Information.

To align a system of elongated particles such as nanofibrils, a pure extensional flow with minimal shear will always be the preferential source of hydrodynamic forcing. As briefly discussedearlier, the reasons for avoiding the shear flow are three: 1) shear can cause flexible polymers and nanofibrils to curl and fold into bundles, as well as generate rotation due to the vorticity; 2) shear can cause stiff particles to flip and rotate intermittently; 3) shear can cause semiflexible particles to flocculate into larger semi‐spherical flocks rotating with vorticity; 4) shear can cause a relative receding motion in the alignment direction between two nearby particles. All these effects are disruptive in the controlled assembly of aligned elongated particles. Furthermore, the perceived shear alignment can be deceiving if observed in the direction of vorticity, as the deformation is planar, and the alignment is far worse when observed in the gradient direction (**Figure** **13** and Videos S1–S3, Supporting Information).

However, for any process‐relevant flow, avoiding shear altogether is not trivial as the dispersion needs to be pushed through tubing and nozzles, and therefore be in contact with the confining walls. Even if an extensional flow can be included by pushing the dispersion through a converging nozzle or needle, shear can still disrupt the system.^[^ ^82^ ^]^ Nevertheless, if the dispersion can be detached from the confining walls, shear can be avoided. This is also why the flow‐focusing technique is superior in terms of creating an aligned system of nanofibrils. The sheath flows both allow the dispersion to detach from the walls and cause the dispersion to stretch with very low influence of shear as it is focused at the center of the channel.

#### From the Fluid Dynamics Perspective

The flow‐focusing technique, however, has several disadvantages from a general processing perspective, as it requires detailed control of the dispersion properties. First, the rheology of the dispersion and effective interfacial tension between the dispersion and sheath fluids will determine the flow regime.^[^ ^83^, ^84^ ^]^ The desired detached core flow for filament spinning is usually referred to as a threading regime. Given the properties of sheath fluid and dispersion, the flow regime could also be dropping (core dispersion forms droplets) or tubing (core dispersion never detaches from the walls perpendicular to sheath flows). Furthermore, running the system at high flow rates, there are hydrodynamic instabilities arising from recirculation bubbles appearing where the sheath flows are rapidly turning 90°.^[^ ^85^ ^]^ All of these instabilities are usually avoidable in syringe‐based processing, where the spinning velocities thus can be much higher.

#### Controlling Timescales

The controlled alignment and assembly with the flow‐focusing technique requires proper control of the timescales in the system.^[^ ^13^, ^17^ ^]^ The general principle is that the nanofibrils should align during spinning and that the transition to an arrested (gel) state through the addition of ions occurs before Brownian rotary diffusion has dealigned the system. Successful assembly thus relies on understanding the following time scales:

1) The timescale for alignment *t*_alignment_. Controlled by flow (rates/geometry).
2) The timescale for Brownian rotary diffusion *t*_diffusion_. Controlled by dispersion.
3) The timescale for gelation *t*_gelation_. Controlled by gel agent, flow, and dispersion.
4) The time the material spends in the assembly section *t*_assembly_. Controlled by flow.

There are several scenarios that can provide control of the assembly of nanofibrils in flow. Firstly, if *t*_alignment_ < *t*_diffusion,_ hydrodynamical forces overcome Brownian motion and align the system. Secondly, if *t*_alignment_ *< t*_gelation_ < *t*_diffusion_ the structure is locked in a gel state before Brownian motion dealigns the system but after hydrodynamic alignment. Thirdly, if *t*_gelation_ < *t*_assembly_, the alignment and gelling is completed within the assembly section of the continuous material production line. Ideally, the time scales would consequently be ordered as: *t*_alignment_ *< t*_gelation_ < *t*_assembly_ < *t*_diffusion_.

Ensuring that *t*_alignment_ is low, typically means that flow rates are high and is usually limited by the flow‐instabilities described above. The time scale *t*_assembly_ can be adjusted by extending the assembly section downstream of the focusing section. However, if the geometry is fixed, the time scale puts an additional limitation on the flow rates. Controlling *t*_gelation_ is trickier as this is both controlled by the diffusivity of type of ions used as a gel agent but also on the cross‐sectional flow geometry determining the distance over which the ions diffuse. It might also be determined by the dispersion, as the type of nanofibrillar network might affect the ion diffusivity.

#### Rotary Diffusion

One of the key aspects in controlling the alignment of nanofibrils is to try to maximize the rotary diffusive time scale *t*_diffusion_. This would both allow us to reach a higher degree of alignment at lower flow rates, as well as maintaining the aligned structure for long enough time for an arrested state to form. The typical time scales of the rotary diffusion of nanofibrils in dilute systems with the widths of a few nanometers and aspect ratios of 100–200 is only a fe w milliseconds. It is clear from recent works that the slowest rotary diffusion time scales in the semidilute cellulose nanofibril dispersions can reach up to a few seconds.^[^ ^82^, ^86^, ^87^ ^]^ The rotary diffusion process is also found to be clearly a multitime scale process reflecting the polydispersity of the length distribution as well as the entanglements and steric hindrance characteristic of a loose network of the longest nanofibrils. The latter part is the main reason for the slow rotary diffusion, allowing alignment of the system at relatively slow flow rates. Therefore, this sets a crucial condition on the dispersion used for spinning, the concentration needs to be carefully chosen given a certain length distribution of nanofibrils. The concentration needs to be low enough to allow for some mobility of the nanofibrils to align with the extensional flow, but high enough for the loose network effects to slow down the rotary diffusion.

### 4.3 Characterization Techniques to Obtain Dispersion Properties Relevant for Filament Spinning

To design an optimal process to produce filaments, careful characterizations of rheological properties as well as nanoscale dynamics and structures during flow and gel formation are needed. Not every dispersion will be suitable for spinning, as small morphological changes to the dispersion during preprocessing (e.g., subtle variations of oxidation and/or homogenization) can completely change the time scales in the system and possibly not even lead to a stable flow in a threading regime. Furthermore, understanding and controlling a dispersion of nanofibrils in flowing systems requires in situ characterization on a nanometer scale. Even though the dynamics and interactions give rise to macroscopic properties, such as orientation distributions and rheology, the ability to characterize in detail what is happening on the sub‐microscopic scale is limited, since this would require a sub‐nanometer imaging resolution and a time‐resolution below a microsecond. Ideally, the measurement should also be noninvasive/noncontact to prevent interference with the system dynamics and the assembly processes. Such techniques are far from realizable in any foreseeable future. It should also be noted that all imaging techniques provide a projected view of reality lacking information on out‐of‐viewing‐plane motion. The solution would be to capture all dynamics using stereographic or tomographic imaging allowing the reconstruction of the 3D motion using simultaneous projections from several viewing angles, which indeed would require a very complicated experimental setup.

#### Microscopic Techniques

Present imaging techniques that can resolve individual nanofibrils are typically restricted to static images of dried samples using either electron microscopy (TEM) or atomic force microscopy (AFM). Cryo‐TEM is an interesting technique to study the structures also in dispersion by quickly freezing the sample,^[^ ^88^ ^]^ which brings us closer in understanding the nanoscale structures in the dispersion, but still does not reveal any dynamics. Although in situ liquid TEM has been demonstrated for certain specific systems,^[^ ^89^ ^]^ it has yet not been demonstrated at a sub‐millisecond level for nanofibrillar systems. In situ characterization using liquid TEM of stained nanofibrils might be a possibility in the near future, although there will be a challenges to achieve sufficient contrast. To get insights into nanoscale interactions, we depend on techniques that captures average quantities, from which qualified guesses are drawn based on models. Examples of such techniques include polarized optical microscopy (POM), rheometry, and scattering techniques (light and X‐rays).

#### Measuring Differences in Refractive Indices

**Figure 14:**
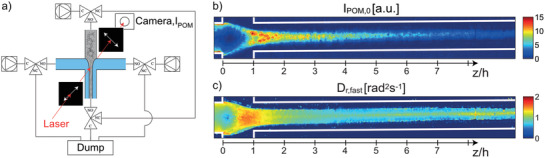
Illustration of the POM flow‐stop experiment demonstrated by Rosén et. al.^[^ ^82^ ^]^ a) The flow‐focusing flow cell is placed between two cross‐polarized linear polarization filters and the intensity of the laser‐illuminated setup is recorded by a camera and illustrated in (b). The flow can be instantly stopped through the switching of three‐way slider valves and the decay of birefringence due to Brownian motion is recorded, allowing to map local rotary diffusion coefficients along the channel, as illustrated in (c). a) Adapted with permission.^[^ ^82^ ^]^ Copyright 2020, The Royal Society of Chemistry.

Alignment of nanofibrils causes the dispersion to become birefringent, a property that can be readily measured using polarized optical microscopy (POM).^[^ ^90^ ^]^ By recording the transmitted light intensity after passing through two crosspolarized filterson each side of the sample, the average alignment of the nanofibrils can be probed. With this technique it is thus possible to study how nanofibrils are aligning when subject to some external force, as well as the collective dealignment caused by Brownian rotary diffusion when any other ordering/aligning force is instantly removed. In a recent demonstration,^[^ ^82^ ^]^ it was shown how rotary diffusion of nanofibrils could be measured in a flow cell by recording the decay of birefringence while switching from a flowing system aligning the nanocellulose through hydrodynamic forces, to an instantaneous stop of the flow (**Figure** **14**). This showed that the rotary diffusion process includes multiple time scales. Subsequent work showed how these different time scales can be attributed to the polydispersity of the dispersion,^[^ ^86^, ^87^ ^]^ where the fastest time‐scales for dealignment relate to short, nonentangled nanofibrils, while the longest timescales rather relate to longer nanofibrils, which are at least partially entangled in a loose network. Using this POM flow‐stop technique, it is thus possible to probe collective dynamics, which can be used to estimate sizes/lengths and interactions of the nanofibrils. It should be noted that birefringence as a phenomenon is dependent on concentration and crystallinity of the material. Given the multitude of factors that contribute to the results in the typical POM flow‐stop experiment, the characterization is not sufficient and other techniques are needed if the results should represent real physical quantities.

A related technique to study differences in refractive index is optical coherence tomography (OCT), which can be used to both study the 3D velocity profile in the flow‐focusing system as well as characterizing the detachment from the walls and transitions between flow regimes,^[^ ^84^ ^]^ which in turn can be very useful to verify the dispersion flow properties and effective interfacial tension.

#### Rheometry

The dynamics and interactions of nanofibrils also affects the work needed to deform a volume of the dispersion, e.g., by shearing forces, which can be measured on a macroscopic scale as a change in apparent viscosity in a rheometer. As discussed earlier, the reorientation of nanofibrils during shearing gives rise to both shear‐thinning and thixotropic behavior, and the apparent viscosity depends on the concentration and nanofibril morphology. By measuring the apparent viscosity at different nanofibril concentrations, the critical “overlap” concentration can be determined.^[^ ^91^ ^]^ At the overlap concentration, the dispersion also gets different viscoelastic properties, which can be measured with oscillating shear rheometry. A dilute dispersion with no nanofibrillar interactions is more “liquid”‐like and has a complex storage modulus lower than the loss modulus. On the contrary, for a semi‐dilute dispersion, which rather consists of a loose network of partially entangled network of nanofibrils, the storage modulus is higher than the loss modulus, and is consequently more “gel”‐like. It should be pointed out that a critical assumption done in shear rheometry is that the entire dispersion is deformed in the same way. However, performing this at high concentrations might lead to large flocks being formed by shearing, or there will be a water layers forming at the walls with strong shear, while the bulk dispersion is not deformed. To verify the velocity profile and detect flock‐formation, the shear rheometry can be combined, e.g., with OCT measurements.^[^ ^92^ ^]^ The mechanistic behavior of the dispersion of nanofibrils measured with rheometry can be used to give us some clues on how the nanofibrils behave in a flowing situation. Still, this is far from enough to know if it will lead to a stable threading regime when spinning in a flow‐focusing process, and will affect spinnability generally.

#### Scattering Techniques

##### Static Scattering Techniques

Even though individual nanofibrils cannot be resolved with dynamic imaging techniques, the way light scatters off the particles can be used to characterize the material further. If a sample is illuminated by a focused beam of light, the intensity of the scattered light on a detector will represent the amplitude of the projected Fourier transform of the illuminated particles. With scattering techniques, it is thus possible to get information about the average shape, orientation and spatial correlations of the nanofibrils. The probed length scales of the dispersion are dependent on the wavelength of light and the angle between the incoming and scattered light, while smaller length scales are being probed at wider angles. Visible light scattering techniques using laser light (SLS/DLS) are normally limited to dilute dispersions and can only probe dynamics on length scales similar to the wavelength. In order to study the process relevant dispersions of cellulose nanofibers at length scales down to the dimensions of single nanofibrils, one would need to resort to small‐angle X‐ray and neutron scattering (SAXS and SANS) techniques.

The most common utilization of SAXS/SANS is to perform static measurements, where time dependence is averaged out. The scattering pattern will be dominated by the average cross‐sectional shape of the nanofibrils as the major dimension is probed at angles too close to the direct beam.

In an isotropic (nonflowing) dispersion, the pattern is also isotropic and described by a single 1D structure function of scattering intensity versus angle (magnitude of scattering vector). By assuming a certain cross‐sectional geometry of the nanofibrils, the average cross‐sectional dimensions can be extracted by curve fitting of the structure function. However, cellulose nanofibers do not represent a system that is easily characterize in this way. First, there is a controversy on the actual cross‐sectional shape of the nanofibrils, even though several SAXS studies favor a ribbon‐shaped nanofibril on average.^[^ ^93^, ^94^ ^]^ Second, a natural system of cellulose nanofibril is very ill‐defined, where nanofibrils vary significantly in lengths and widths given not only the biological origin but also extraction processes. This means that an appropriate model for the curve fitting should include polydispersity. The nonuniformness of the dispersion also leads to a naive scattering curve, almost without any distinct features. Fitting a complicated function (model) to a featureless curve, will lead to high uncertainties with respect to the extracted parameters. It is therefore reasonable to question the accuracy in determining the cross‐sectional dimensions from SAXS/SANS. However, there are no better methods available to characterize these properties of the nanofibrils in the dispersed liquid state.

Given the fact that nanofibrils also give rise to spatial correlations if packed close enough, it has also been found that aggregation/gelation of nanofibrils can be investigated using static SAXS/SANS. Even though the scattering intensity at angles corresponding to cross‐sectional dimensions remains largely the same, it has been demonstrated that the formation of a VAS of nanofibrils with NaCl is distinguished by higher scattering intensity at the smallest angles.^[^ ^93^ ^]^ This feature can be satisfactorily explained by a given correlation length of nanofibril segmental aggregates, which gives an indication of the relative degree of interfibrillar connections in the VAS. To study dilute dispersions of nanofibrils has on the other hand been proven very challenging as this requires mass concentrations well below 0.05wt%. The problem is that the raw scattering signal is dominated by the background (window material and solvent), resulting in a weak scattering signal when subtracting the scattering signal from the background. One possibility would be to study dilute systems using SAXS in the water window, i.e., with soft X‐rays in the range of 2.33–4.40 nm, where water is practically invisible to the X‐rays, which should lead to an increasing contrast between particles and solvent.

**Figure 15:**
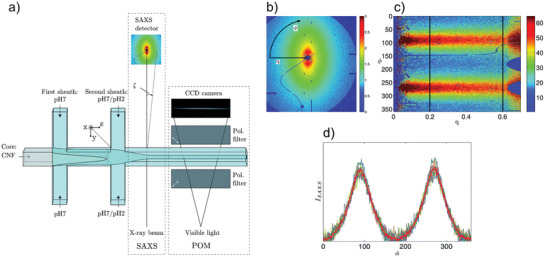
Example of how small‐angle X‐ray scattering (SAXS) and polarized optical microscopy (POM) can be used to study the flow‐induced alignment of CNF in situ during the spinning process.^[^ ^15^ ^]^ a) Focusing an X‐ray beam in the channel gives rise to an anisotropic scattering detector image due to the extensional flow of nanofibrils. The scattering image in (b) is described in polar coordinates and normalized in each *q*‐range to get the diffractogram in (c). d) The average angular profile in a certain *q*‐range (describing length scales between the average width and length of the CNF) is a representation of the orientation distribution function (ODF) of the projected angle in the plane perpendicular to the beam. a–d) Adapted with permission.^[^ ^15^ ^]^ Copyright 2015, The Royal Society of Chemistry.

For the case of alignment by hydrodynamic forces, the scattering pattern also becomes nonisotropic. The normalized scattering intensity in azimuthal direction at a given scattering angle (corresponding to length scales between particle length and width) represents the projected orientation distribution function (ODF) of the illuminated nanofibrils in the plane perpendicular to the beam.^[^ ^95^ ^]^ An example of how this is done is illustrated in **Figure** **15**.^[^ ^90^ ^]^ The orientational information is not dependent on the exact cross‐sectional shape of the nanofibrils and can thus be extracted without any fitting. However, again, since the dispersion of cellulose nanofibers is polydisperse, it does contain particles that are not aligned as well as shorter nanofibrils where the Brownian rotary diffusion is too fast.

**Figure 16:**
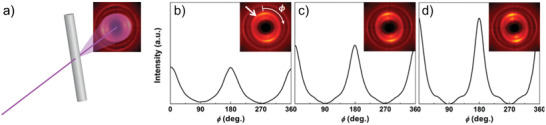
Example from of how wide‐angle X‐ray scattering (WAXS) can be used to study the alignment of CNF inside a filament in situ during wet stretching in the setup illustrated in Figure 1.^[^ ^34^ ^]^ The wet filament is stretched from an initial length *L*_1_ to a length *L*_2_, while focusing the X‐ray beam on the filament and collecting the scattered photons on a detector, illustrated in (a). b–d) Azimuthal intensity profiles of the (200) Bragg reflection (white arrow in (b) revealing the nanofibril orientation distribution for different stretching ratios SR = *L*_2_/*L*_1_ − 1. b) SR = 0; c) SR = 0.16; d) SR = 0.28. b–d) Adapted with permission.^[^ ^34^ ^]^ Copyright 2014. American Chemical Society.

Using hard X‐rays (wavelength typically below 1 Å), the internal structure of the individual nanofibrils could be determined by just considering the scattering at higher angles. With wide‐angle X‐ray scattering (WAXS), the crystal configuration inside the nanofibrils can be determined given the scattering peaks corresponding to the crystal lattice. The projected ODF of nanofibrils can thus be derived through the normalized scattering intensity of these Bragg reflections in azimuthal direction.^[^ ^95^ ^]^ **Figure** **16** shows an example of how WAXS was utilized to study in situ the nanofibril alignment during wet‐stretching of CNF filaments.^[^ ^34^ ^]^

All previous WAXS characterizations of nanocellulose are relying on measurements of almost dry samples as water has very strong scattering at these angles even for semidilute dispersions. If this background subtraction problem can be overcome, there is a possibility using WAXS for different in situ studies of dispersions where processes might affect the internal cellulose structure. As it is difficult to obtain a good contrast between nanofibrils and solvent with X‐rays as, the scattering intensity is only dependent on the electron density in the sample, an alternative is to use neutron scattering techniques where the contrast can be modified by changing the isotopic configuration of the solvent.

##### How to Quantify Alignment and Orientational Order

One of the most used quantities to quantify the orientation of nanofibrils is their “degree of alignment” or “degree of orientational order” along the flow direction. This quantity has many definitions and often spans between a value of 0 (isotropic) and 1 (fully aligned). It is commonly determined from an anisotropic SAXS/WAXS detector image I(**q**), whereby subsequent conversion to polar coordinates, normalization, and averaging provides an orientation distribution function (ODF) Ψ_χ_ of the projected angle χ on a plane perpendicular to the X‐ray beam.^[^ ^95^ ^]^ Most often, the degree of alignment is evaluated through an order parameter based on the 2nd Legendre polynomial ${\langle P_{2}\rangle }_{\chi }=\langle P_{2}(\cos\chi )\rangle =\langle \frac{1}{2}(3\cos\chi -1)\rangle$ (sometimes called Hermans orientation parameter) through

(3) $$S_{\chi }^{3\text{D}}=\int_{0}^{\pi }\Psi_{\chi }\;\left(\frac{3}{2}\cos^{2}\chi -\frac{1}{2}\right)\sin\chi \text{d}\chi$$

with normalization

(4) $$1\;=\int_{0}^{\pi }\Psi_{\chi }\;\sin\chi \text{d}\chi$$

The normalization with the sin χ‐term is introduced to treat the projected angle on a 2D plane as a polar angle in spherical coordinates.^[^ ^95^ ^]^ The true 3D order parameter $S_{\phi }^{3\text{D}}$ based on the actual spherical polar angle ϕ can be calculated by knowing the ODF Ψ*_ϕ_* in the same way. If the system is known to have cylindrical symmetry, which generally is an appropriate assumption both in during spinning and in the filament, the ODF Ψ*_ϕ_* can be calculated from Ψ_χ_.^[^ ^95^ ^]^ A more suitable choice to describe alignment in the detector plane would be to use an order parameter based on the projected angle according to

(5) $$S_{\chi }^{2\text{D}}=\int_{0}^{\pi }\Psi_{\chi }\;(2\cos^{2}\chi -1)\text{d}\chi$$

with normalization

(6) $$1\;=\int_{0}^{\pi }\Psi_{\chi }\;\text{d}\chi$$

Other definitions of the degree of alignment include the orientation index^[^ ^17^, ^96^ ^]^

(7) $$f_{\text{c}}=\frac{\pi -\text{FWHM}}{\pi }$$

where FWHM is the full‐width half‐maximum of Ψ_χ_. It is also possible to provide an alignment factor AF based directly on the *I*(**q**) without the intermediate conversion to an ODF.^[^ ^97^ ^]^

All these scalar parametrizations ($S_{\chi }^{3\text{D}}$, $S_{\chi }^{2\text{D}}$,$S_{\phi }^{3\text{D}}$, *f*_c_, AF) describing the anisotropy of the system have the property of being 0 for an isotropic system and 1 for a fully aligned system. However, for any degree of anisotropy in between, these parameters are never the same. As an example, this makes it impossible to know if $S_{\chi }^{3\text{D}}=\;0.5$ implies more “aligned” than *f*_c_ = 0.4.

The reduction of dimensionality from a 2D ODF to a 1D scalar parameter of course means a huge loss of information, as there can be an infinite number of ODFs that can result in the same 1D parameter. Furthermore, one must be aware of the fact that the 2D ODF from SAXS/WAXS only provides the perceived (projected) alignment from one particular viewing direction. The most obvious problem is to study systems, which do not have any cylindrical symmetry around the axis of alignment, e.g., a system aligning due to shear, which is a planar 2D deformation. As discussed previously, this leads to a completely different perceived alignment if the system is observed in the flow‐gradient plane or in the flow‐vorticity plane (cf. Figure 13). Only describing the alignment in the 2D plane can also lead to interesting implications even in a cylindrically symmetric system.^[^ ^95^ ^]^ For example, in a system aligned through increasing uniaxial extension, the true 3D order parameter $S_{\phi }^{3\text{D}}$ can show almost perfect alignment, even though the measured projected order parameter is $S_{\chi }^{3\text{D}}\approx 0.6$.

Both problems, i.e., the loss of information through dimensionality reduction and the dependence of viewing direction, must be properly considered whenever comparing the average orientations between different experiments, or ever comparing experiments to theoretical/numerical models. Although the most proper comparison would be directly of the 2D ODF Ψ_χ_, 1D scalar parametrizations are still very useful. Apart from measuring the degree of alignment with an order parameter, it would already be a huge improvement if orientation distributions would also be compared through a 1D parameter describing the approximate shape of the ODF. A suggested shape parameter γ, based on the 4th Legendre polynomial 〈*P*_4_〉_χ_, is defined as^[^ ^95^ ^]^

(8) $$\gamma =\frac{\log\left(\frac{\log{\left\langle P_{4}\right\rangle }_{\chi }}{\log{\left\langle P_{2}\right\rangle }_{\chi }}\right)}{\log2}$$

which can be used to determine if the ODF is “smoother” (γ < 1) or “pointier” (γ > 1).

##### Dynamic Scattering Techniques

With coherent light and fast detectors, it is also possible to obtain the time‐dependent loss of spatial correlation, and thus diffusive dynamics, through photon correlation spectroscopy (PCS). With laser light sources, this has been done extensively through the technique called dynamic light scattering (DLS), but this of course is limited by sample concentration and scattering angles as mentioned before. Recent advances at synchrotron radiation sources and free electron lasers, including increasing coherence and faster detectors, have enabled the use of dynamic X‐ray scattering through X‐ray photon correlation spectroscopy (XPCS).^[^ ^98^ ^]^ This technique extracts the instantaneous spatial correlations of nanofibrils from the interference pattern on the detector. Ideally, when comparing two images in a sequence with a defined lag time, the change in spatial correlation can be determined. This in essence is a measurement of the collective diffusive dynamics of nanofibrils. However, the technique still suffers from several drawbacks, which makes it very difficult (if not impossible) to utilize XPCS for dispersions of cellulose nanofibers. Firstly, since the dispersions are very dilute (typically around 99.7 wt% water), the scattering contrast is very low. Second, the relevant dynamics of individual nanofibrils is very fast, typically on a time scale of microseconds or lower, which means that the exposure time of each XPCS image also needs to be sub‐microsecond. Thus, with low scattering contrast, there is almost no information in an image to be correlated. Finally, the technique relies on the same set of nanofibrils being illuminated for a longer time. Since the highly energetic X‐rays are very destructive to any biobased samples, the noninvasiveness of the method can be rightly questioned. Another possible way of determining dynamic properties of radiation sensitive colloidal dispersions is to utilize X‐ray speckle visibility spectroscopy (XSVS).^[^ ^99^ ^]^ Given the early development stage of these methods relying on coherent X‐rays, it is highly possible that future advances will enable the usage of XPCS/XSVS to study dispersions of nanofibrils in general and cellulose nanofibers specifically.

#### Numerical Methods to Understand Nanofibril Behavior in Flows

The experimental techniques available to study nanofibril systems only give us some pieces of the puzzle toward understanding the complete picture, specifically with respect to dynamic behavior. Given the difficulty to characterize nanoscale dynamics, one has to rely on theoretical models and numerical simulations to develop model systems that fit the experimental evidence.

At the most fundamental level, there are molecular dynamics (MD) simulations that can be used to predict motion and interactions of individual atoms, molecules, and supramolecular structures. Today, there are massive simulations that can simulate millions of atoms to describe different systems on the time scale of a few milliseconds.^[^ ^100^ ^]^ Although this might be enough to simulate interactions on the scale of the nanofibril cross‐section of a few nanometers, it is still not sufficient to simulate the interactions of several nanofibrils in an entangled network. Other coarse‐grained methods could aid in this, where Brownian motion is simulated without tracking the motion of individual atoms.^[^ ^101^ ^]^

To be able to predict the macroscopic coupling between fluid dynamics and nanofibril dynamics in systems relevant to spinning, other methods need to be implemented. The flow of a cellulose nanofibril dispersion in a flow‐focusing geometry has been accurately simulated by matching rheology and effective interfacial tension with experimental measurements.^[^ ^84^ ^]^ This provides a very good tool for predicting local velocity gradients in the flow. Neglecting translational diffusion of the dispersed nanoparticles and further neglecting the two‐way coupling to the flow, the rotational dynamics can be simulated using different theoretical approaches by knowing the velocity gradients along the streamlines. Assuming the particles to be Brownian ellipsoids, the particle rotation can be simulated,^[^ ^102^ ^]^ which was done and compared with flows of cellulose nanocrystals.^[^ ^81^ ^]^ To get statistical information of the particle orientation, the time needed to perform many single particle simulations might be unreasonable. Therefore, the evolution of the orientation distribution can be directly simulated with a Smoluchowski equation using the a well‐known theoretical framework.^[^ ^66^ ^]^ Although specifically derived for polymer dynamics, the theory can be used to capture the main features of the aligning nanofibrils.^[^ ^103^ ^]^ Still it is clear that the models are incomplete and need to be improved to account for both the polydispersity and entanglement effects.^[^ ^87^ ^]^

## 5 Suggestions for Further Developments of Nanocellulose Spinning Processes

To develop spinning processes for fabrication of high performance nanocellulose‐based fibers (aiming at mechanical performance and functionality) requires the understanding and control of morphology and interactions in the nanofibrillar system. Still, much of the research published characterizes the starting dispersion and the final fiber, but rarely the process steps in between. Additionally, the material characterization often relies on methods and models that were initially developed for other purposes, making the interpretation confusing and sometimes misleading. For example, for a system of nearly spherical particles or folded proteins, it makes sense to discuss the hydrodynamic radius measured with DLS or a radius of gyration in SAXS. The same quantities can be measured in a nanocellulose dispersion, but it is less obvious how to relate them to physical properties of the nanofibrillar network.

Scattering techniques are extremely powerful for studying monodisperse, highly ordered systems, but using them for our complex nanofibrillar system often results in a catch‐22 problem, where a complicated polydisperse system typically gives rise to very smeared distributions of experimental quantities (time scales, lengthscales, etc.) resulting in scattering curves without any prominent features. A physically correct model should include all the relevant variables but fitting a complicated model with many variables to a simple curve creates a large uncertainty in the fitted results of the variables. The true power of scattering techniques, however, is that they can be easily used for in situ process characterization. Being able to characterize the nanofibril orientation distribution and static/dynamic structure factors directly in the flow, which can provide us with much more useful information about the dispersion to improve the processes.

Crucial quantities for developing and controlling the spinning process are

- Flow‐induced alignment and spatial structure in different velocity gradients (shear, extension, etc.). Can be obtained with in situ scanning SAXS experiments.
- Rheology and effective interfacial tension. Can be obtained with rheometry and OCT.
- Mobility and dynamics of the nanofibrils. Can be obtained with the POM flow‐stop technique and possibly XPCS.

Similarly, although we have been emphasizing alignment and assembly processes, there are also needs to quantify:

- Alignment of nanofibrils during drying at different gel drawing conditions as well as in the final dried material. Can be obtained with WAXS.
- Mechanical properties of the material (strength, stiffness, toughness, etc.). Can be obtained with tensile measurements.

With respect to spinning filaments from cellulose nanofibers, the goal is to find efficient and quantitative means of predicting the final material properties based on known dispersion qualities and the specific process, e.g., flow‐focusing. Ultimately, there is a need to be able to answer the following questions:

- Can the dispersion of nanofibrils be modified to maximize filament properties given a certain spinning process?
- Can the spinning process be adapted to maximize filament properties given a certain dispersion of nanofibrils?

The described characterization techniques POM, rheometry, and X‐ray scattering can experimentally provide important collective features of particles in a dispersion including birefringence, resistance to deformation and the Fourier‐transformed amplitude of the electron density distribution in the dispersion. All these features are the result of the dynamics and interactions of individual nanofibrils, but it is still largely a guessing game when it comes to understanding the system on the nanofibril level. There are too many unknown parameters that contribute to the experimental results, which makes it difficult to determine the most important system parameters. This in turn makes it difficult to deduce an optimal processing condition for a given dispersion of nanofibrils.

Instead of relying on experimental results to make guesses about the smaller scales of the system using a top‐down approach, we would propose developing our scientific knowledge based on a methodology where bottom‐up simulations are iteratively adapted to fit calculated experimental observables. Even a simulation model that does not match the experimental results can be used to rule out certain unknown parameters. The model should thus be as simple as possible while still capturing the relevant physics/chemistry in the system. This still relies on a clever design of simulations and experiments in a way where it is possible to easily distinguish between two or more hypotheses. More importantly, this approach relies on a highly quantitative comparison of the measurables and the same conceptual framework when comparing results with different research groups.

This algorithmic approach to improve simulations given experimental results could possibly be automized by spanning a large variety of system parameters and eventually teaching the computer to directly interpret the experimental data through machine learning and artificial intelligence. By further designing experiments where system variables can be easily tuned and a large amount of data can be collected, it might be possible to let the automated analysis identify reliable empirical constitutive equations useful for optimizing nanofibril dispersions and processes.

It is our firm belief that the combination of simulation and experiment will provide much greater insight into the nature of dispersed nanofibril dynamics compared to an approach only relying on experimental characterization. Of course, this approach requires more careful experimental set‐ups to allow for in situ characterization using, e.g., SAXS at a synchrotron facility. It also requires a high level of collaboration and cross‐functional teams with expertise in numerical simulations, fluid dynamics, characterization techniques, machine learning as well as chemical synthesis. Still, this framework is believed to be essential to progress both in terms of scientific breakthrough, to find out the true nature of dispersed cellulose nanofibers, and engineering innovations, to develop new processes to continuously assemble nanostructured cellulose materials with tunable properties on an industrial scale.

## Conflict of Interest

The authors declare no conflict of interest.

## Supporting information

Supporting Information

Supplemental Video 1

Supplemental Video 2

Supplemental Video 3
