# Supplementary material for: Elucidating the Opportunities and Challenges for Nanocellulose Spinning
Source: Adv Mater. 2020 Aug 23;33(28):2001238. doi: 10.1002/adma.202001238 (PMC11468825; doi:10.1002/adma.202001238)
Supplement: Supplementary file 1 — Supporting Information [file ADMA-33-2001238-s001.pdf]

© 2020 Wiley-VCH GmbH

ADVANCED MATERIALS

Supporting Information

for *Adv. Mater.*, DOI: 10.1002/adma.202001238

Elucidating the Opportunities and Challenges for Nanocellulose Spinning

*Tomas Rosén, Benjamin S. Hsiao, and L. Daniel Söderberg**

Supporting information

Elucidating the Opportunities and Challenges for Nanocellulose Spinning

Dr. Tomas Rosén 1, Prof. Benjamin S. Hsiao 2 and Prof. L. Daniel Söderberg 1.

Address line 1, Wallenberg Wood Science Center, KTH Royal Institute of Technology, S-100 44, Stockholm, Sweden

Address line 2, Chemistry Department, Stony Brook University, Stony Brook, NY 11794-3400, USA

E-mail: dansod@kth.se

Table S1. A selected set of results with focus on the development of mechanical performance. Specific acronyms used in the table: CNF=non-modified CNF, TCNF=Tempo CNF, CMC CNF= Carboxymethylated CNF).

Reference	Nanocellulose	Strength [MPa]	Stiffness [GPa]	Strain [%]	Comment
Wet spinning					
Iwamoto et al. (2011)[1]	TCNF-wood	332	19.1	3.1	
	TCNF-tunicate	406	18.9	5.6	
Walther et al. (2011)[2]	TCNF-wood	275	22.5	4	
Lundahl et al. (2016)[3]	TCNF-wood	297	21.3	2.8	
	CNF-wood	326	15.5	6.9	
	CNF-wood	223	12.7	8.4	
Mohammadi et al. (2017)[4]	CNF-wood	328	18.8	12.1	
Wang et al. (2019)[5]	TCNF-wood	207	12	3.6	Ethanol
	TCNF-wood	236	15	4.6	Acetone
	TCNF-wood	344	17	4.5	Ionic cross-linking using $CaCl_2$
	TCNF-wood	329	18	2.7	HCl
Lundahl et al. (2018)[6]	CNF-wood	70	2.1	6	Coaxial-spinning
Yao et al. (2017)[7]	TCNF-bacterial	198	12	4.5	
	TCNF-bacterial	248.6	16.4	3.8	Post-spinning stretching
	TCNF-bacterial	357.5	22.9	2.3	Stretching and Fe^{3+} cross-linking
Vuoriluoto et al. (2017)[8]	TCNF-wood	237	16	2.8	
	TCNF-wood	127	11.6	1.3	With benzophenone, UV cross-linking.
Geng et al. (2017)[9]	TCNF-jute	268.7	22.8	~2	
	TCNF-jute	369.8	28.9	~2	PAE cross-linking
Torres-Rendon et al. (2014)[10]	TCNF-wood	118	8.2	8.3	
	TCNF-wood	289	33.7	1.6	Post-spinning stretching
Kafy et al. (2017)[11]	TCNF-wood	249.7	16.8	9.2	
	TCNF-wood	383.3	21.1	6.6	Post-spinning stretching
Kim et al. (2019)[12]	TCNF-wood	492.6	24.3	11.8	
	TCNF-wood	543.1	37.5	3.7	Post-spinning stretching
Wet spinning (flow-focusing)					
Håkansson et al. (2014)[13]	CMC CNF-wood	490	17.6	6.4	
Mittal et al. (2017)[14]	TCNF-wood	830	53	6	
Mittal et al. (2018)[15]	TCNF-wood	~1200	~70	5 - 6	50% RH
	TCNF-wood	1320	82	1 - 2	20% RH
	TCNF-wood	1430	70	5 - 6	BTCA cross-linking and 50% RH
Nechyporchuk et al. (2019)[16]	CMC CNF-wood	443	17.2	7.4	
Mittal et al. (2019)[17]	CMC CNF-wood	1010	57	5 - 6	
	CMC CNF-wood	770	43	-	
	CMC CNF-wood	834	39	8.0	Ionic cross-linking

Table contnd.

Reference	Nanocellulose	Strength [MPa]	Stiffness [GPa]	Strain [%]	Comment
Interfacial complexation					
Toivonen et al. (2017)[18]	TCNF-wood	204	15	7.6	
	TCNF-wood	240	23	4.1	PDADMAC as cation
Grande et al. (2017)[19]	TCNF-bagasse	220	22		
Zhang et al. (2018)[20]	TCNF-wood	153	8.4	4.9	
Dry spinning					
Hooshmand et al. (2015)[21]	CNF- banana rachis	222	12.6	~3	
Shen et al. (2016)[22]	CNF-wood	145	-	-	
	CNF-wood	220	-	-	Stretching on-line
Ghasemi et al. (2017)[23]	CNF-wood	~90	~5	-	Dried at room-temperature
	CNF-wood	~100	~6.5	-	Dried at 430 deg.
Wet spinning (mixtures)					
Lundahl et al. (2018)[6]	CNF-wood	29.9	0.9	5.5	With cellulose acetate
	CNF-wood	69.5	2.1	6.2	With guar gum
Li et al. (2015)[24]	TCNF-wood	442.4	34.1	2.0	With GO, cross-linking ($CaCl_2$)
Wet spinning (flow-focusing mixtures)					
Mittal et al. (2017)[25]	TCNF-wood	980	54	10	With recomb. spider silk
Nechyporchuk et al. (2019)[16]	CMC CNF	330	18	5.4	With CNC
Dry spinning (mixtures)					
Hooshmand et al. (2017)[26]	CNF-wood	150	8.5	5.1	
	CNF-wood	179	11.3	4.5	Cold-drawn
	CNF-wood	260	15.0	5.7	HEC and cold-drawn

Video S1 (Shear_lambda10.avi)

Dynamics of initially randomly oriented spheroidal particles of aspect ratio 10 in shear flow according to the theory by Jeffery (1922)[27]; (left) 3D view; (middle) view of flow-gradient plane; (right) view of flow-vorticity plane; spheroids are performing an intermittent tumbling motion with highest projected alignment in the flow-gradient plane.

Video S2 (Shear_extension10.avi)

Dynamics of initially randomly oriented spheroidal particles of aspect ratio 10 in uni-axial extensional flow according to the theory by Jeffery (1922)[27]; (left) 3D view; (middle

and right) view of the two different extension-compression planes; spheroids are instantly aligning in the extensional direction.

Video S3 (Shear_compression10.avi)

Dynamics of initially randomly oriented spheroidal particles of aspect ratio 10 in uni-axial compressional flow according to the theory by Jeffery (1922)[27]; (left) 3D view; (middle) view of compression-extension plane; (right) view of the extensional plane; spheroids become randomly oriented in the extensional plane (perpendicular to compression).

References

[1] S. Iwamoto, A. Isogai, T. Iwata, Biomacromolecules 2011, 12, 831.

[2] A. Walther, J. V. I. Timonen, I. Diez, A. Laukkanen, O. Ikkala, Adv. Mater. 2011, 23, 2924.

[3] M. J. Lundahl, A. G. Cunha, E. Rojo, A. C. Papageorgiou, L. Rautkari, J. C. Arboleda, O. J. Rojas, Scientific Reports 2016, 6.

[4] P. Mohammadi, M. S. Toivonen, O. Ikkala, W. Wagermaier, M. B. Linder, Scientific Reports 2017, 7.

[5] L. Wang, M. J. Lundahl, L. G. Greca, A. C. Papageorgiou, M. Borghei, O. J. Rojas, Scientific Reports 2019, 9.

[6] M. J. Lundahl, V. Klar, R. Ajdary, N. Norberg, M. Ago, A. G. Cunha, O. J. Rojas, ACS Appl. Mater. Interfaces 2018, 10, 27287.

[7] J. Yao, S. Chen, Y. Chen, B. Wang, Q. Pei, H. Wang, ACS Appl. Mater. Interfaces 2017, 9, 20330.

[8] M. Vuoriluoto, H. Orelma, M. Lundahl, M. Borghei, O. J. Rojas, Biomacromolecules 2017, 18, 1803.

[9] L. Geng, B. Chen, X. Peng, T. Kuang, Mater. Des. 2017, 136, 45.

[10] J. G. Torres-Rendon, F. H. Schacher, S. Ifuku, A. Walther, Biomacromolecules 2014, 15, 2709.

[11] A. Kafy, H. C. Kim, L. Zhai, J. W. Kim, L. Van Hai, T. J. Kang, J. Kim, Scientific Reports 2017, 7.

[12] H. C. Kim, D. Kim, J. Y. Lee, L. Zhai, J. Kim, International Journal of Precision Engineering and Manufacturing-Green Technology 2019, 6, 567.

[13] K. M. O. Håkansson, A. B. Fall, F. Lundell, S. Yu, C. Krywka, S. V. Roth, G. Santoro, M. Kvick, L. P. Wittberg, L. Wågberg, L. D. Söderberg, Nature Communications 2014, 5.

[14] N. Mittal, R. Janson, M. Widhe, T. Benselfelt, K. M. O. Håkansson, F. Lundell, M. Hedhammar, L. D. Söderberg, Acs Nano 2017, 11, 5148.

[15] N. Mittal, F. Ansari, V. K. Gowda, C. Brouzet, P. Chen, P. T. Larsson, S. V. Roth, F. Lundell, L. Wågberg, N. A. Kotov, L. D. Söderberg, Acs Nano 2018, 12, 6378.

[16] O. Nechyporchuk, K. M. O. Hakansson, K. V. Gowda, F. Lundell, B. Hagstrom, T. Kohnke, Adv. Mater. Technol. 2019, 4.

[17] N. Mittal, T. Benselfelt, F. Ansari, K. Gordeyeva, S. V. Roth, L. Wågberg, L. D. Söderberg, Angewandte Chemie International Edition 2019, 58, 18562.

[18] M. S. Toivonen, S. Kurki-Suonio, W. Wagermaier, V. Hynninen, S. Hietala, O. Ikkala, Biomacromolecules 2017, 18, 1293.

[19] R. Grande, E. Trovatti, A. J. F. Carvalho, A. Gandini, Journal of Materials Chemistry A 2017, 5, 13098.

[20] K. Zhang, H. Liimatainen, Small 2018, 1801937.

[21] S. Hooshmand, Y. Aitomaki, N. Norberg, A. P. Mathew, K. Oksman, ACS Appl. Mater. Interfaces 2015, 7, 13022.

[22] Y. Shen, H. Orelma, A. Sneck, K. Kataja, J. Salmela, P. Qvintus, A. Suurnaekki, A. Harlin, Cellulose 2016, 23, 3393.

[23] S. Ghasemi, M. Tajvidi, D. W. Bousfield, D. J. Gardner, W. M. Gramlich, Polymers 2017, 9.

[24] Y. Li, H. Zhu, S. Zhu, J. Wan, Z. Liu, O. Vaaland, S. Lacey, Z. Fang, H. Dai, T. Li, L. Hu, Npg Asia Materials 2015, 7.

[25] A. Abbadessa, P. Oinonen, G. Henriksson, BioResources 2018, 13, 7606.

[26] S. Hooshmand, Y. Aitomaki, L. Berglund, A. P. Mathew, K. Oksman, Composites Science and Technology 2017, 150, 79.

[27] G. B. Jeffery, Proceedings of the Royal Society of London. Series A, Containing papers of a mathematical and physical character 1922, 102, 161.
